# The development of media truth discernment and fake news detection is related to the development of reasoning during adolescence

**DOI:** 10.1038/s41598-025-90427-z

**Published:** 2025-02-26

**Authors:** Marine Lemaire, Steeven Ye, Lorna Le Stanc, Grégoire Borst, Mathieu Cassotti

**Affiliations:** 1 Université Paris Cité, LaPsyDÉ, CNRS, Paris, F-75005 France; 2 Institut Universitaire de France, Paris, F-75005 France; 3 IPSOS France, Global Science Organisation, Paris, France

**Keywords:** Fake news, Media truth discernment, Illusory truth effect, Dual process theory, Adolescents, Psychology, Human behaviour

## Abstract

The spread of online fake news is emerging as a major threat to human society and democracy. Previous studies have investigated media truth discernment among adults but not among adolescents. Adolescents might face a greater risk of believing fake news, particularly fake news that is shared via social media, because of their vulnerabilities in terms of reasoning. In the present study, we investigated (1) the development of media truth discernment and the illusory truth effect from adolescence to adulthood and (2) whether the development of media truth discernment and the illusory truth effect are related to the development of reasoning ability. To accomplish this task, we recruited 432 adolescents aged 11 to 14 years as well as 132 adults. Participants were asked to rate the perceived accuracy of both real and fake news headlines. Participants were exposed to half of the news items before entering the rating phase. Finally, participants completed the Cognitive Reflection Test (CRT). Media truth discernment (i.e., the difference between participants’ ratings of fake and real news) developed linearly with increasing age, and participants rated familiarized headlines as more accurate than novel headlines at all ages (i.e., the illusory truth effect). Finally, media truth development (but not the illusory truth effect) was related to the development of reasoning abilities with increasing age. Our findings highlight the urgent need to improve logical thinking among adolescents to help them detect fake news online.

**Supplementary Information:**

The online version contains supplementary material available at 10.1038/s41598-025-90427-z.

The spread of online fake news is emerging as a major threat to human society and democracy^1^. A paradigmatic example of this threat for modern society lies in the events of January 6th, 2021, when a mob broke into the U.S. Capitol in Washington, DC, to protest the results of the U.S. presidential election; these events were fueled by fake news regarding massive electoral fraud^2^. The 2018 presidential election in Brazil provided yet another example of the growing tendency for the spread of fake news via social media to influence the outcomes of elections^3^. The term fake news is commonly defined as fabricated content that is created with the intent to deceive people^4^. Fake news is typically circulated online to manipulate public opinion and influence events, including the election of Donald Trump in 2016^5,6^, COVID-19 vaccinations^7^, belief in climate change^8^ and, recently, the war in Ukraine^9^.

Although considerable efforts have been invested to increase our understanding of the psychological mechanisms involved in news media evaluation^10,11^, little is known about the ability of adolescents to detect fake news or the psychological processes underlying the development of such ability. This research gap is highly surprising given that nearly 95% of adolescents in the U.S. own a smartphone by the age of 13^12^; in addition, adolescents spend on average 1.27 h per day on social media^13^. Finally, adolescents receive their news more frequently from social media and YouTube than from major news organizations, and 78% of this group report that it is important to follow the major events occurring at a given time^14^. In this context, we investigated the process by which the ability to discern fake news from real news develops from early adolescence to young adulthood in relation to the development of analytical thinking.

Among adults, previous research originally linked belief in fake news with motivations related to partisanship: people with high analytical reasoning abilities tend to use their cognition to rationalize beliefs in news that aligns with their partisan inclinations and to be skeptical of news that is discordant with those inclinations^15^. Another account of why people fall for fake news focuses on the field of reasoning, particularly with regard to dual-process theories. According to these theories, human cognition can be characterized by a distinction between autonomous, intuitive (System 1) processes and deliberative, analytic (System 2) processes^16,17^. People tend to rely on System 1 processes, leading to systematic errors in response to simple reasoning problems^18^. For instance, in the Cognitive Reflection Test (CRT)^18^, participants are typically asked to determine the cost of a ball after being told that “*A ball and a bat cost 110 euros*,* the bat costs 100 euros more than the ball*”. While the logical answer is 5 euros (the response according to System 2), most people erroneously state that the ball costs 10 euros (the response according to System 1). According to dual-processes theories, people who rely more heavily on System 1 processes might be at greater risk of experiencing difficulty in their attempts to discern fake content from real content. Consistent with this hypothesis, an increasing number of studies have identified an association between performance on the CRT and the ability to discern fake news from real news^11^, and analytical thinking seems to play a more prominent role in fake news beliefs than in real news beliefs^19,20^. Importantly, people who are more reflective (i.e., those who rely more on System 2) are better at discerning between real news and fake news (i.e., media truth discernment) regardless of whether the news in question is consistent or inconsistent with their ideology and beliefs^19–22^.

Media truth discernment appears to be influenced not only by people’s reflectivity levels but also by contextual factors. In particular, repetition of content increases the perceived accuracy of such content, i.e., the so-called “illusory truth effect” (for reviews, see^23,24^). For instance, repetition increases the perceived accuracy of trivia statements^25^, opinion statements^26^, and rumors^27^ even when these statements are highly implausible^28^ or contradict participants’ prior knowledge^29^. The illusory truth effect has also been observed in the domain of news content, with repeated fake news and real news both being perceived as more accurate than novel types of such news^30^. This effect has also been reported in children as young as five years old^31^.

While an increasing number of studies have documented the psychological mechanisms underlying media truth discernment among adults, to date, no study has investigated the development of media truth discernment during adolescence. Adolescence may constitute a period of vulnerability with regard to media truth discernment for three main reasons. First, the ability to overcome biases in reasoning (System 1 processes) while relying on analytical thinking (a System 2 process) develops from preadolescence to middle adolescence^32–35^. Second, the ability to detect conflicts between System 1 and System 2 processes in the context of reasoning tasks, which is a prerequisite for overcoming biases in reasoning, continues to develop from 13 years of age to young adulthood^36,37^. Third, while adolescents are confident in their ability to evaluate the credibility of information, they can fail to detect the presence of conflicts of interest^38^ or to identify sponsored content^39^.

In the present study, we investigated: (1) the development of media truth discernment and the illusory truth effect from adolescence to adulthood, and (2) whether the development of media truth discernment and the illusory truth effect are related to the development of reasoning abilities. We hypothesized that:

1. If analytical thinking is associated with media truth discernment^19,20^, and if analytical thinking develops linearly between the ages of 8 and 15^32^, then media truth discernment would also increase linearly during early adolescence, sustained in part by the development of reasoning abilities. Specifically, we expected that the difference between the true and fake news ratings increased with age.
2. If both children and adults experience the illusory truth effect^30,31^, then adolescents should also be sensitive to this effect and rate repeated news as more accurate than novel ones (no expectation concerning the developmental trend).
3. If analytical thinking (measured with the CRT) is fundamental to media truth discernment and fake news detection^19,20^, then CRT performance should mediate the relation between age and media truth discernment and fake news detection, and to a lesser extent, real news detection.

To accomplish this task, we recruited 432 adolescents aged 11 to 14 years as well as 132 young adults. Participants were asked to rate the perceived accuracy of both real and fake news headlines on a 4-point Likert scale (ranging from 1 = “not accurate at all” to 4 = “very accurate”). Participants were exposed to half of the news items before rating their accuracy. We computed participants’ levels of media truth discernment (i.e., the difference in the ratings they provided between real and fake news) and the illusory truth effect (i.e., the difference in the ratings they provided between familiarized and nonfamiliarized news)^30^. Participants also completed the CRT.

## Results

### The development of media truth discernment

**Fig. 1:**
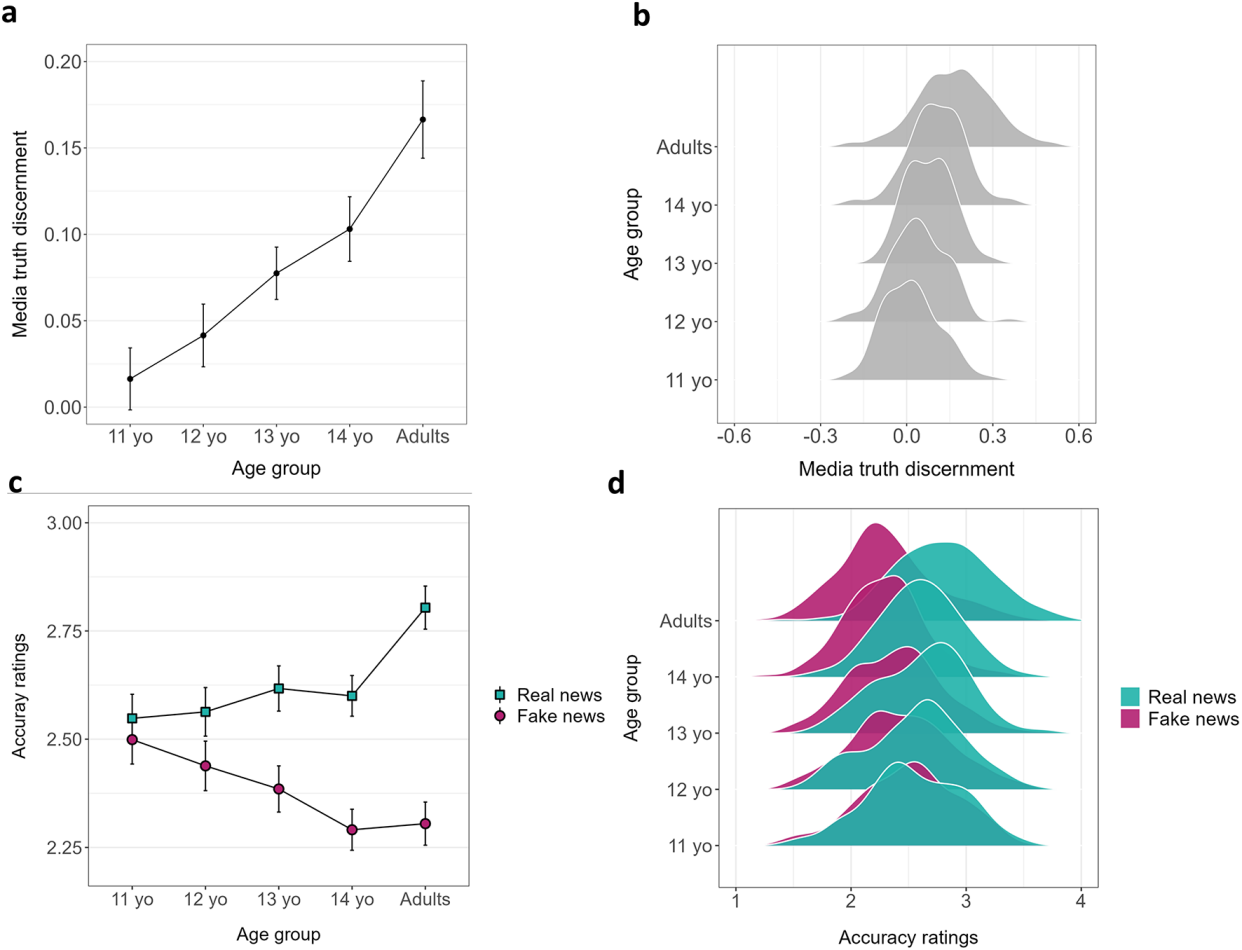
Development of the ability to evaluate the veracity of news from young adolescence to adulthood. (**a**) Mean media truth discernment scores (higher scores reflect greater accuracy with regard to discriminating between real and fake news) as a function of age group. (**b**) Distribution of the mean media truth discernment scores for each age group. (**c**) Mean accuracy ratings concerning real and fake news items as a function of age group (scored using a Likert scale ranging from 1: “not accurate at all” to 4: “very accurate”). (**d**) Distribution of the mean accuracy ratings pertaining to real and fake new items for each age group. Error bars indicate 95% confidence intervals.

Figure 1 shows the average media truth discernment scores (Fig. 1a and b) as well as the average accuracy ratings for real and fake headlines (Fig. 1c and d) across the five age groups (for descriptive statistics, see SI – Tables S1 and S2). To investigate the development of media truth discernment across age groups, we performed a one-way ANOVA (type III sum of squares) with media truth discernment score as the dependent variable and age group (five levels: 11-years-old, 12-years-old, 13-years-old, 14-years-old, and adults) as the independent variable. The analysis revealed a significant main effect of age group (*F*(4, 559) = 38.50, *p* < .001, *η²p* = .22, 95% CI [0.17, 1.00], SI – Table S3), indicating that media truth discernment significantly differed across age groups and improved linearly with age (significant linear trend using polynomial contrast: *M*_estimate_ = 0.11, *SE*_estimate_ = 0.009, *t*(559) = 12.00, *p* < .001; SI – Table S3). The partial eta squared (*η²p* = .22) indicates a large effect size, suggesting that age group explained a substantial part of the variance in media truth discernment. Post-hoc comparisons (all p-values for post-hoc comparisons were corrected using the Holm method) revealed two significant developmental shifts: adults were significantly more efficient at discerning real from fake news than 14-year-olds (*t*(559) = 4.70, *p* < .001, *d* = 0.40, 95%CI [0.23, 0.56]), and 13-year-olds were significantly more efficient at this task than 12-year-olds (*t*(559) = 2.57, *p* = .03, *d* = 0.22, 95%CI [0.05, 0.38]) (Fig. 1a and b; SI – Table S3). The effect size for the difference between adults and 14-year-olds (Cohen’s *d* = 0.40) indicates a medium effect, while the effect size between 13- and 12-year-olds (Cohen’s *d* = 0.22) suggests a small difference between the two groups.

To test the effect of age on real news and fake news, a three-way ANOVA (2 [veracity: real news, fake news] × 2 [repetition: novel, repeated] × 5 [age group: 11-years-old, 12-years-old, 13-years-old, 14-years-old, and adults]) was conducted with accuracy ratings as the dependent variable using type III sum of squares (SI – Table S4). Note that the three-way interaction was not significant and unrelated to any of our hypotheses (*p* = .16). Importantly, the analysis revealed a significant and large interaction between veracity and age group (*F*(4, 559) = 38.43, *p* < .001, *η²p* = .22, 95% CI [0.16, 1.00]). Post-hoc comparisons (Holm-corrected) showed that all age groups statistically significantly rated real news as more accurate than fake news (all *p*’s < 0.001), with the exception of 11-year-old adolescents (*t*(559) = 1.66, *p* = .09, *d* = 0.07, 95%CI [-0.01, 0.15]; SI – Table S4). These findings suggest that the ability to distinguish between fake news and real news begins to emerge as early as 12 years of age (Fig. 1c and d).

Partial correlation analysis (with age as control variable; SI – table S5) showed that the conflict items included in the CRT were associated with media truth discernment scores (Pearson’s correlation: *r* = .23, *p* < .001) representing a small effect size, as well as fake news detection (Pearson’s correlation: *r* = − .16, *p* < .001), a small effect size. In contrast, the no-conflict items were only weakly associated with media truth discernment scores (Pearson’s correlation: *r* = .10, *p* < .01) (for the developmental pattern of the CRT, see SI – Fig. S1 and Table S6).

**Fig. 2:**
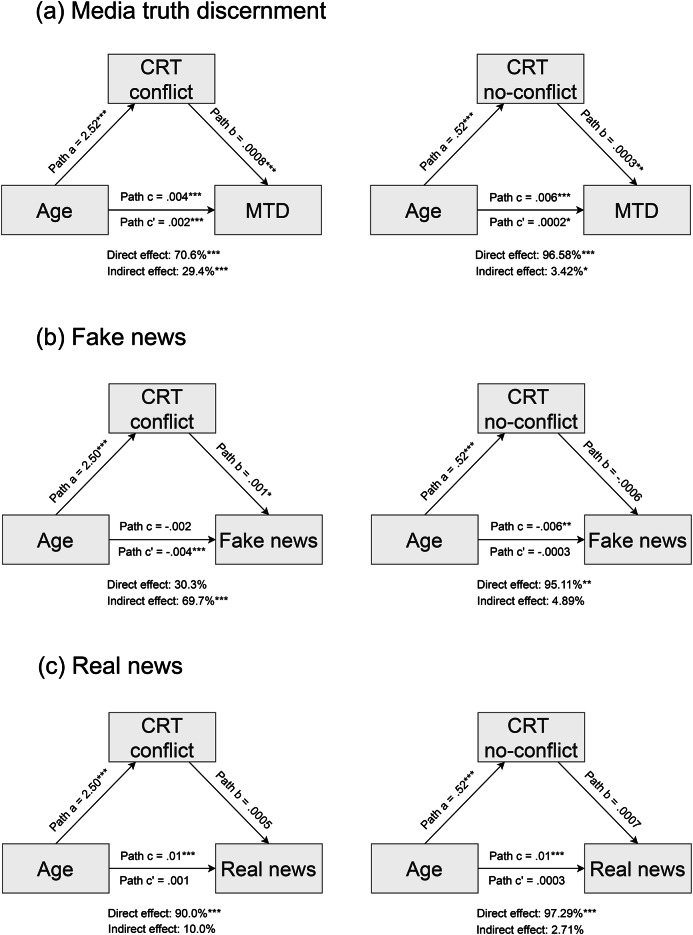
Mediation analysis including CRT conflict and no-conflict scores as mediators, age as the independent variable and (**a**) media truth discernment, (**b**) fake news items and (**c**) real news items as the dependent variables. Path c reflects the direct effect, while path c’ reflects the indirect effect. The percentage of mediation indicates the percentage of the total effect for which the indirect and direct effects account (using the “medmod” module in Jamovi 2.3.24 software). *p < .05, ** *p* < .01, *** *p* < .001.

The relationship between age and media truth discernment was strongly mediated by the conflict items (simple mediation analysis, indirect path: c’ = 0.001, *p* < .001, indirect effect = 29.3%, *p* < .001; SI – table S7), as well as, to a lesser extent, by the no-conflict items included in the CRT (simple mediation analysis, indirect path: c’ = 0.0002, *p* = .02, indirect effect = 3.42%, *p* = .02; Fig. 2a; SI – table S7). Interestingly, different patterns emerged depending on whether fake news or real news were used as outcome variable instead of media truth discernment scores in the mediation analysis (Fig. 2b and c). The relationship between age and fake news accuracy ratings was fully mediated by the conflict items (simple mediation analysis, indirect path: c’ = -0.004, *p* < .001, indirect effect = 69.7%, *p* < .001; SI – table S8) but not by the no-conflict items of the CRT (simple mediation analysis, indirect path: c’ = − 0.0004, *p* = .30, indirect effect = 4.89%, *p* = .30; SI – table S8). However, the relationship between age and real news accuracy ratings was not mediated by the conflict items (simple mediation analysis, indirect path: *p* = .20; SI – table S9), nor by the no-conflict items (simple mediation analysis, indirect path: *p* = .26; SI – table S9). Identical analyses restricted to novel news (SI - Tables S10 to S17, figure S2 and S3) and to repeated news items (SI -Tables S18 to S25, fig. S4 and S5) exhibited similar results.

### The development of the illusory truth effect

**Fig. 3:**
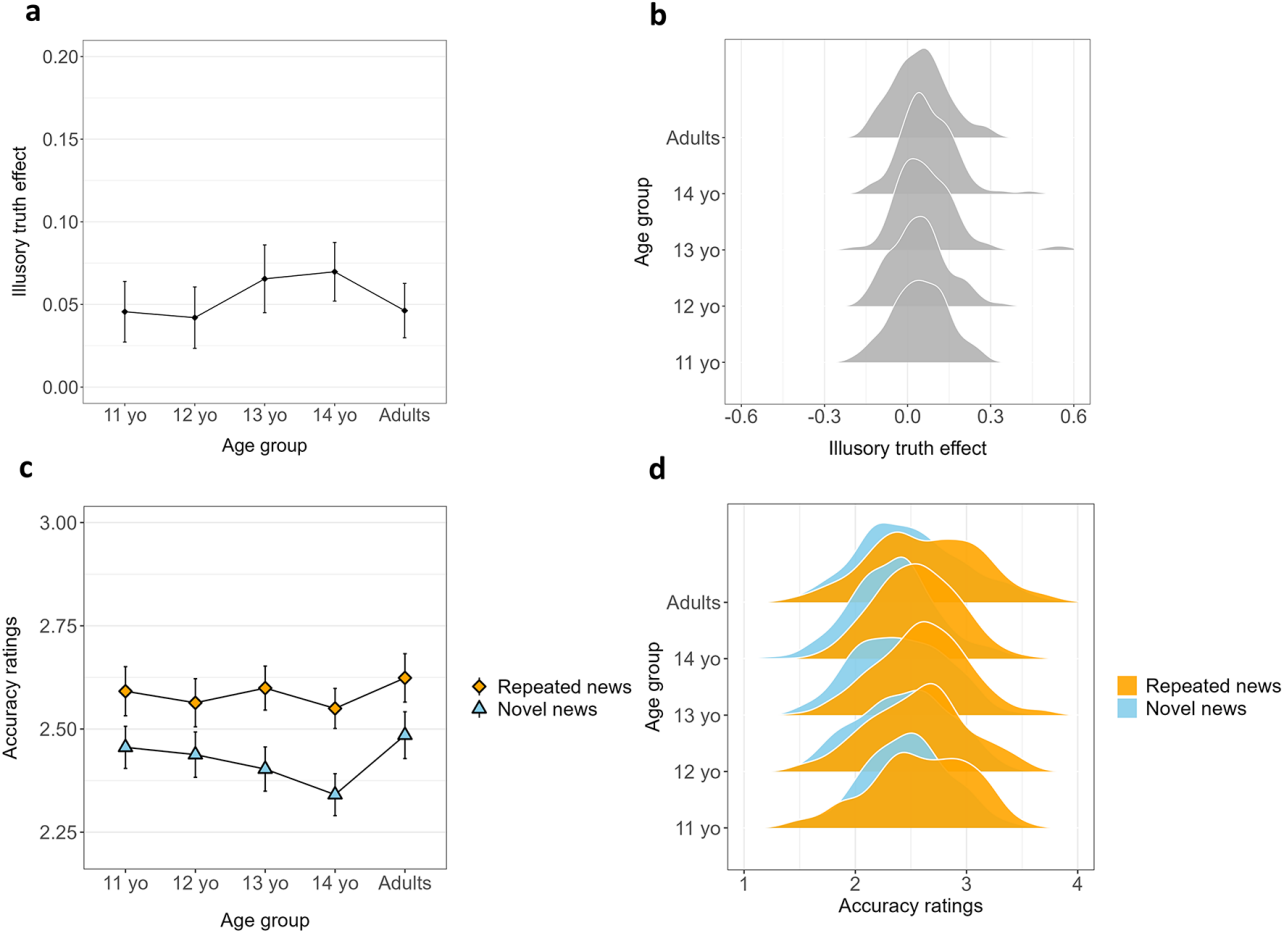
Development of the illusory truth effect from young adolescence to adulthood. (**a**) Mean illusory truth effect scores (higher scores reflect greater sensitivity to repetition) as a function of age group. (**b**) Distribution of mean illusory truth effect scores for each age group. (**c**) Mean accuracy ratings pertaining to novel and repeated news items as a function of age group (as scored on a Likert scale ranging from 1: “not accurate at all” to 4: “very accurate”). (**d**) Distribution of the mean accuracy ratings concerning novel and repeated news items for each age group. Error bars indicate 95% confidence intervals.

Figure 3 illustrates the average illusory truth scores (Fig. 3a and b) and the average accuracy ratings for repeated and novel headlines (Fig. 3c and d) across the five age groups (for descriptive statistics, see SI – Tables S26 and S27). To investigate the development of the illusory truth effect across age groups, we conducted a one-way ANOVA (type III sum of squares) with the illusory truth effect score as the dependent variable and age group (five levels: 11-years-old, 12-years-old, 13-years-old, 14-years-old, and adults) as the independent variable. The analysis revealed no significant effect of age group on the illusory truth effect score (*F*(4, 559) = 1.82, *p* = .12; Fig. 3a and b, SI – Table S28). This result is corroborated by the interaction between age group and repetition in the previously described three-way ANOVA (also SI – Table S4), which was not significant (*p* = .12). The illusory truth effect was similar across the five age groups, with all five groups identifying repeated news headlines as more accurate than novel headlines (main effect of repetition: *F*(1, 562) = 164.78, *p* < .001, *η²p* = .23, 95% CI [0.18, 1.00]; Fig. 3c and d). The effect size (η² = 0.23) suggests a large effect of repetition on the accuracy ratings of news headlines. Interestingly, this repetition effect differed between real and fake news headlines (interaction between veracity and repetition: *F*(1, 562) = 11.08, *p* < .001, *η²p* = .02, 95% CI [0.00, 1.00]). A larger illusory truth effect was observed for fake news (*t*(559) = 16.57, *p* < .001, *d* = 0.70, 95% CI [0.61; 0.79]) compared to real news (*t*(559) = 13.23, *p* < .001, *d* = 0.56, 95% CI [0.47; 0.65]). The small *η²* (0.01) suggests a minimal effect size for the interaction, indicating that while the difference in the illusory truth effect between real and fake news is statistically significant, its practical significance is relatively small. Partial correlation analysis (controlled for age) indicated that the illusory truth effect scores were not significantly associated with media truth discernment scores or with the conflict and no-conflict items included in the CRT (Pearson’s correlation: all *p*’s > 0.08; SI – Table S5).

## Discussion

The aim of the present study was to (1) explore the development of media truth discernment and the illusory truth effect from adolescence to adulthood and (2) investigate whether the development of media truth discernment and the illusory truth effect are related to the development of reasoning abilities. We found that media truth discernment abilities improved linearly from 11 years of age to adulthood, with the ability to discriminate between fake and real news headlines emerging at 12 years of age. Of course, the fact that younger adolescents exhibit poorer media truth discernment does not necessarily imply that they share more false news via social media. Indeed, judging the veracity of information is not systematically related to sharing intentions^40^, and elderly people actually share seven times more fake news via social media than do young adults^41^. Our findings are consistent with the results of previous studies of media literacy and the assessment of news credibility, with adolescents exhibiting poorer media literacy abilities^38^ and poorer abilities to assess digital news credibility^39^. Consistent with dual process theories of media truth discernment^11^, we found that performance on the CRT was related to media truth discernment among both adolescents and adults. Thus, adolescents who are more reflective (i.e., those who rely more on System 2) exhibit better media truth discernment, as has been reported among adults^19–21^. This result is also in line with previous findings showing that children with higher cognitive reflection are better at judging and verifying statements about science, independently of age^42^.

Moreover, we provided the first evidence to suggest that the increased ability to discern real news headlines from fake news headlines that develops between 11 years of age and adulthood is partially mediated by individuals’ increased ability to engage in analytical thinking. Supplementary analyses revealed that performance on the CRT mediates the effect of age on fake news ratings but not on real news ratings, thus suggesting that analytical thinking might be critical for developing the ability to detect fake news but that other processes might support the development of the ability to detect real news. Overall, our results support the hypothesis that overcoming cognitive biases and engaging in analytical thinking could be one process lying at the root of media truth discernment during adolescence but that other processes, such as general knowledge and/or media literacy, might also be involved in the task of identifying real news in particular.

In contrast to media truth, the illusory truth effect (i.e., the tendency of repeated news to be perceived as more accurate than novel news) was already observed at age 11 and remained stable from adolescence to adulthood. Our findings replicate and extend the results of previous studies by showing that the illusory truth effect is observed not only among children^31^ and adults^30,43^ but also among adolescents. In addition, the repetition effect seems to be slightly stronger for fake news than for real news, as has been reported in previous studies^44,45^. Finally, we found no association between CRT scores and the illusory truth effect or between media truth discernment and the illusory truth effect. Thus, the illusory truth effect does not seem to be related to analytical thinking, unlike media truth discernment. Moreover, these results complement previous findings that the illusory truth effect is not influenced by differences in cognitive style or traits^46^. The illusory truth effect is most likely to be the result of the increased fluency produced by the repetition of the same information, which in turn serves as a cue for judging the veracity of the information presented^47,48^ (however alternative account exists for the illusory truth effect^49^).

One limitation of the present study is that the same news headlines were presented to both adolescents and adults; thus, general knowledge could play a role in the increased ability to discern fake news from real news observed from adolescence to adulthood. We cannot rule out the possibility that general knowledge plays a role in the developmental pattern reported in this research. Nonetheless, (a) we selected news headlines that focused on topics that were familiar to adolescents, and (b) analytical ability mediated the effect of age on media truth discernment, thus providing evidence against the claim that general knowledge per se can lie at the root of the developmental pattern reported in this research. Another limitation is the relatively narrow age range of participants (11 to 14 years of age). Although this allowed us to examine developmental trends during early to mid-adolescence, the small age differences may have limited the ability to observe more pronounced age-related differences. Future studies with a broader age range could provide more insights into the developmental trajectory of media truth discernment from early adolescence to young adulthood. Furthermore, we did not explicitly assess participants’ familiarity with the news headlines presented, which could have influenced their judgments. Although we selected news stories that were not overly recent and were not related to major worldwide events, future research could investigate the role of familiarity more directly, for instance by assessing familiarity in a pretest or by including it as a control variable. This could help clarify the extent to which familiarity influences media truth discernment. However, when we examined the repeated news headlines (those that were familiar to participants through the design), we observed similar developmental effects, suggesting that familiarity did not have a major influence on the developmental patterns of media truth discernment in this study. Although our results corroborate the literature on dual process theory, the use of a 4-item CRT may not fully capture the variability associated with analytical reasoning, both in adolescents and adults. We recommend that future research on this topic use a longer version of the CRT, such as the CRT-D^50^, and include non-numerical items^51^ to better capture individual differences in analytical thinking.

In conclusion, the present study provides the first evidence that media truth discernment develops from early adolescence to young adulthood, a process which is partially due to the development of analytical thinking abilities; it also indicates that the illusory truth effect impacts media truth judgments at all ages. Given that adolescents at 11 years of age do not distinguish between real news and fake news despite the fact that most of these individuals already own a smartphone, it is vital to design educational interventions to improve media truth discernment that are based in part on attempts to foster analytical thinking and reduce the illusory truth effect.

## Materials and methods

### Participants

We recruited 599 participants for this study. Thirty-five participants were excluded because they reported having checked the accuracy of the news headlines online. The final sample (*N* = 564) included 432 adolescents in the 6th grade (*N* = 110, *M*_age_ = 11.2 ± 0.48 years), 7th grade (*N* = 108, *M*_age_ = 12.3 ± 0.45 years), 8th grade (*N* = 109, *M*_age_ = 13.1 ± 0.54), or 9th grade (*N* = 105, *M*_age_ = 14.3 ± 0.46) as well as 132 adults (*M*_age_ = 27.1 ± 5.16). The proportions of males and females within each of the five age groups were equivalent (*X*^2^ (4, *N* = 564) = 3.80, *p* = .433) (Supplementary Information – Table S29). The study was approved by the institutional review board (IRB) of the University Paris-Cité (CER UPC − 00012023-90). The research was performed in adherence to national and international standards that regulate the involvement of human participants. Parental informed consent of adolescents was collected before the experiment.

### Media truth discernment task

**Fig. 4:**
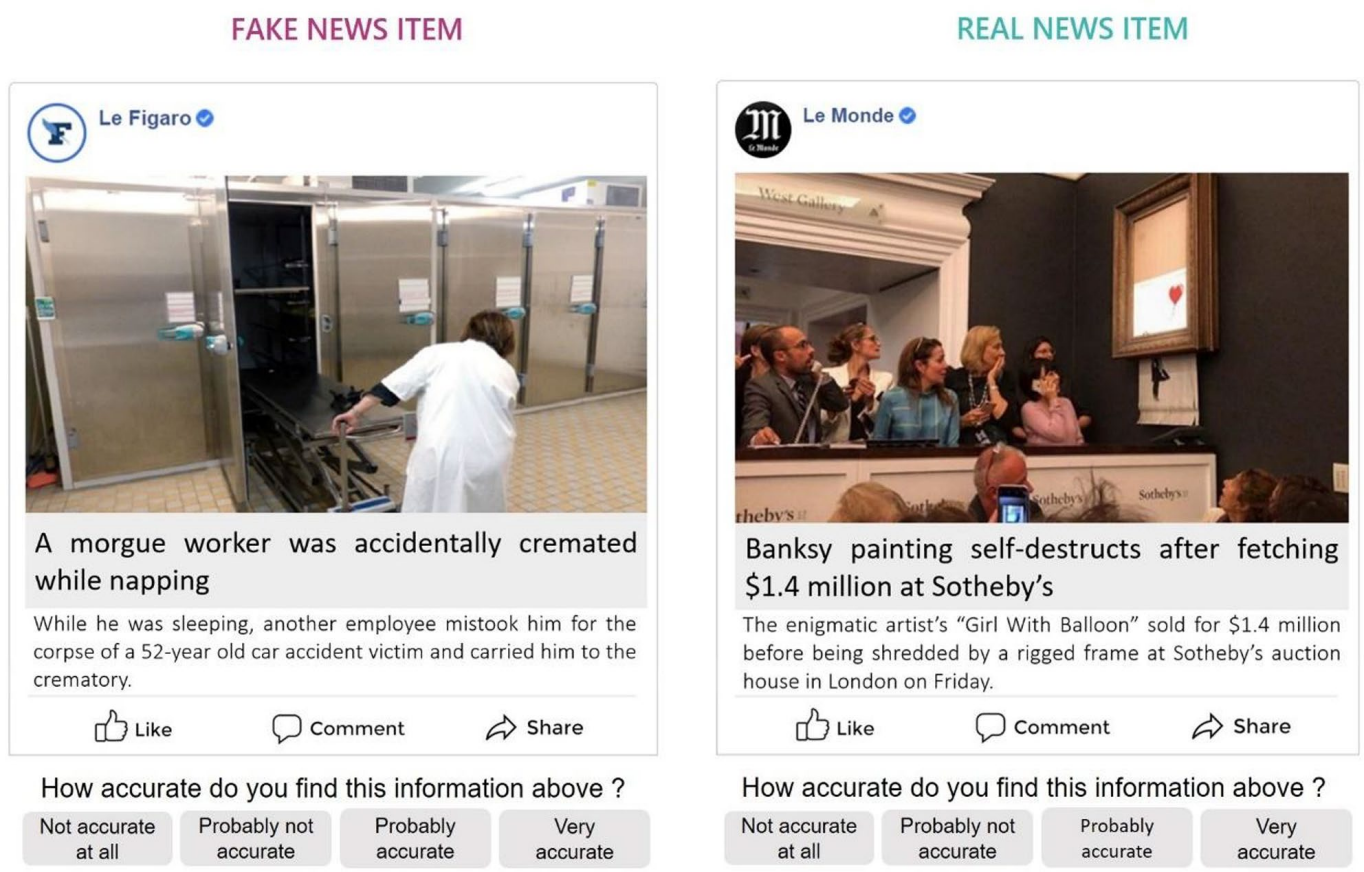
Example of news items used in the experiment; the item on the left is a fake news headline, while the item on the right is a real news headline. The items presented below were translated into English.

We selected 28 true news headlines drawn from mainstream news outlets that featured no factual errors as well as 28 false headlines drawn from fact-checking websites, such as factuel.afp.com, that had been identified as fake (Fig. 4). The procedure to select news headlines was similar to the one used in previous studies^52^. Although we did not explicitly assess the familiarity of these headlines with the participants, we ensured that the true news selected were published at least a year prior testing the participants and were not related to major recent worldwide events, to minimize the likelihood of participants already being familiar with the content. Given that we investigated the development of the ability to evaluate content (title, catchphrase, image) than the ability to determine the source trustworthiness associated with the headline, we removed the source originally associated with the headline to control for its potential effect on the veracity judgments^53,54^. To experimentally control the effect of source trustworthiness, half of the real news and fake news items were paired with either a trustworthy or an untrustworthy news media. The number of words did not vary between the fake and real news items in terms of their titles (*p* = .57, SI - Table S30) or catchphrases (*p* = .58, SI - Table S31). In the *exposure phase*, participants were asked to rate the interestingness of 28 of the news headlines (14 true and 14 false headlines, which were counterbalanced across participants) on a 4-point Likert scale (ranging from 1 = “not interesting at all” to 4 = “very interesting”). After the *exposure phase*, we asked participants to self-evaluate their levels of achievement in different school disciplines (i.e., the *distractor phase*). In the *judgment phase*, participants assessed the accuracy of all 56 news headlines (28 of which were presented during the *exposure phase*) on a 4-point Likert scale (ranging from 1 = “not accurate at all” to 4 = “very accurate”). We computed the media truth discernment score for each participant by subtracting the participant’s average score with regard to false headlines from the corresponding score with respect to real headlines and dividing by 3^20^. Scores that were close to 1 reflected a high level of media truth discernment ability, while scores that were close to 0 indicated a low level of media truth discernment ability. We also computed the illusory truth score as the difference between the average accuracy score for news items that were presented during the exposure phase and novel items, divided by 3. A score that was closer to 0 reflected lower sensitivity to prior exposure to the news headlines.

### Cognitive reflection test

Participants completed four items that were adapted from the original version of the CRT^18^. Two of these items were conflict items (e.g., *“If it takes 5 minutes for 5 machines to make 5 widgets*,* how long would it take for 100 machines to make 100 widgets?”)* in which the System 1 response interfered with the System 2 response, while two other items were no-conflict items (e.g., *“If it takes 10 machines 10 minutes to print 10 books*,* how many machines would it take to print 100 books in 10 minutes?”*), in which the System 1 and System 2 processes provided the same response. For each item, we coded 1 for correct answer and 0 for incorrect responses, or for a no-response. We then computed the accuracy rate for each participant separately on each condition.

### Analysis plan

The data were analyzed using Jamovi version 2.3.24, employing the ‘medmod’ package for mediation analysis. Effect sizes and their confidence intervals were on computed on R Studio, software version 4.3.3^55^ using *effect size* package^56^ All ANOVAs were conducted using type III sum of squares, and the modalities of each analysis are detailed below. The development of media truth discernment was assessed through a one-way ANOVA (type III sum of squares) with media truth discernment scores as the dependent variable and age group (five levels: 11-years-old, 12-years-old, 13-years-old, 14-years-old, and adults) as the independent variable. Polynomial contrasts were used to test the presence of a linear trend in media truth discernment, and post-hoc comparisons with Holm correction^57^ were conducted to assess differences between age groups. To test the existence of the illusory truth effect across all age groups, we conducted a one-way ANOVA (type III sum of squares) with illusory truth effect scores as the dependent variable and age group (five levels) as the independent variable. To evaluate the effect of veracity (real news vs. fake news) and repetition (repeated vs. novel news) on accuracy ratings across the five age groups, we performed a mixed-design ANOVA (type III sum of squares) with veracity and repetition as within-subject factors and age group (five levels: 11-years-old, 12-years-old, 13-years-old, 14-years-old, and adults) as a between-subject factor. The three-way structure (2 × 2 × 5) was analyzed, and the veracity effect was evaluated through the interaction between veracity and age group, followed by post-hoc comparisons with Holm correction to assess group differences. For the repetition effect, we analyzed the interaction between repetition and age group, as well as the main effect of repetition, with specific post-hoc comparisons corrected using Holm’s method. Finally, to examine the role of analytical thinking in the development of media truth discernment and the illusory truth effect, we conducted partial correlations, controlling for age as a continuous variable, using Pearson’s correlations. Additionally, to examine whether CRT (a measure of analytical thinking) mediates the observed effects, we conducted mediation analyses using the ‘medmod’ package for Jamovi, with a bootstrap estimation method for standard errors using 1,000 samples. Material and data are available on the Open Science Framework at https://osf.io/ex8rk/?view_only=cf57e03bf1a0426e89d5d47f4f420515.

## Electronic supplementary material

Below is the link to the electronic supplementary material.

Supplementary Material 1

## Data Availability

Material and data are available on the Open Science Framework at https://osf.io/ex8rk/?view_only=42c1d21e3de74137be6ea74c339c4a4d.
