# Supplementary material for: The development of media truth discernment and fake news detection is related to the development of reasoning during adolescence
Source: Sci Rep. 2025 Feb 26;15:6854. doi: 10.1038/s41598-025-90427-z (PMC11865587; doi:10.1038/s41598-025-90427-z)
Supplement: Supplementary file 1 — Supplementary Material 1 [file 41598_2025_90427_MOESM1_ESM.docx]

**Supplementary Information**

**The development of media truth discernment and fake news detection is related to the development of reasoning during adolescence**

**This PDF file includes:**

Figures S1 to S5

Tables S1 to S31

Fig. S1. Accuracy rates (%) of CRT conflict and no-conflict items as a function of age groups. Error bars indicate 95% confidence intervals.


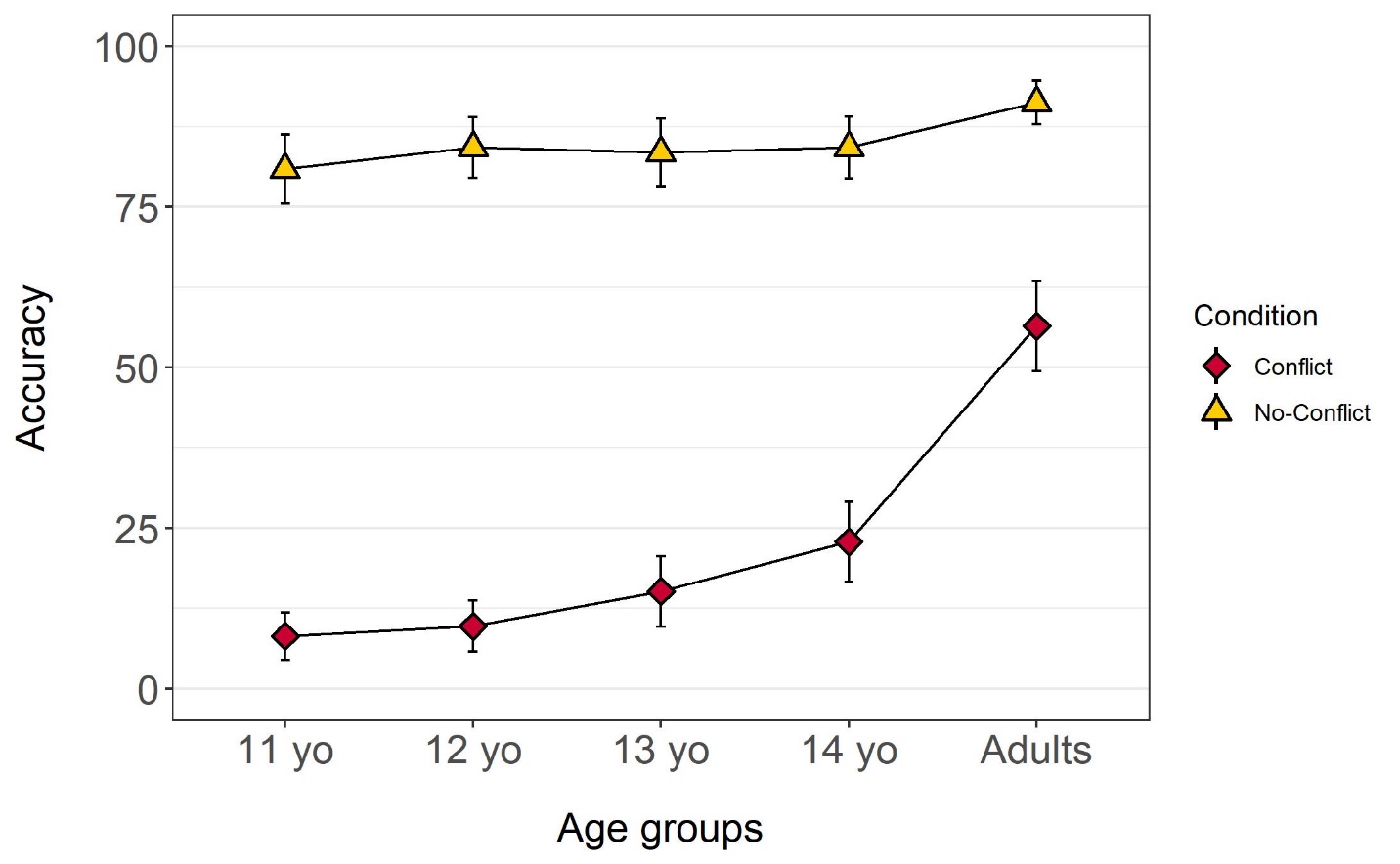
Fig. S2. Development of the ability to evaluate the veracity of news from young adolescents to adults, restricted to novel news items. (A) Mean accuracy ratings concerning real and fake news items as a function of age group (scored using a Likert scale ranging from 1: “not accurate at all” to 4: “very accurate”). (B) Distribution of the mean accuracy ratings pertaining to real and fake new items for each age group. (C) Mean media truth discernment scores (higher scores reflect greater accuracy with regard to discriminating between real and fake news) as a function of age group. (D) Distribution of the mean media truth discernment scores for each age group. Error bars indicate 95% confidence intervals.


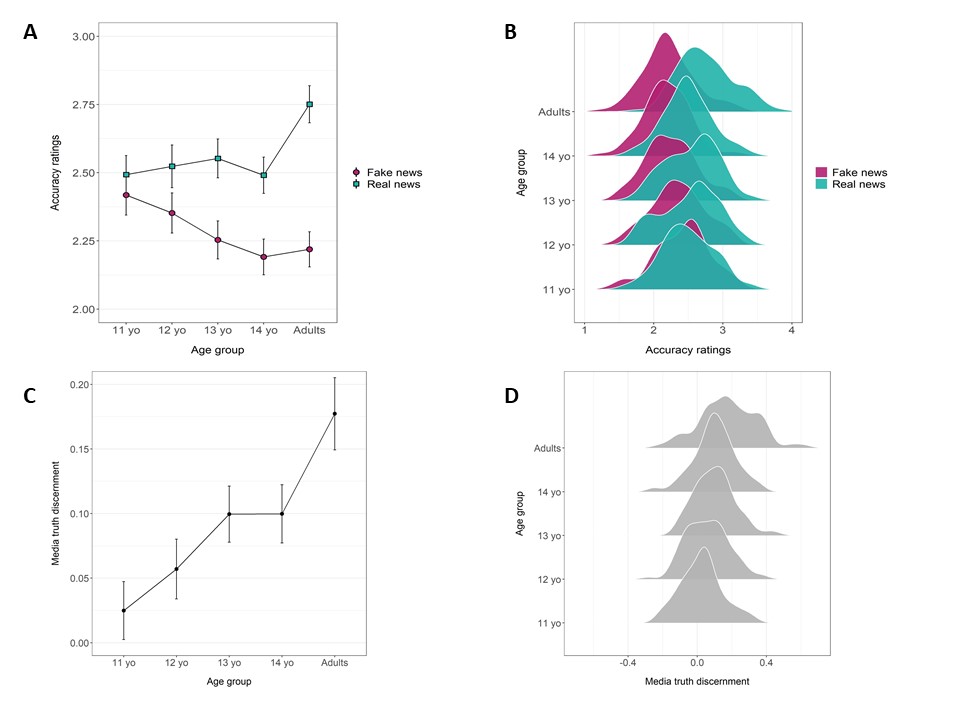


Fig. S3. Mediation analysis restricted to novel news items, including CRT conflict and no-conflict scores as mediators, age as the independent variable and (A) media truth discernment, (B) fake news items and (C) real news items as dependent variables. Path c reflects the direct effect, while path c’ reflect the indirect effect. The percentage of mediation indicates the percentage of the total effect for which the indirect and direct effects account (using the “medmod” module in Jamovi 2.3.24 software). Note. * p < .05, ** p < .01, *** p < .001

**(A) media truth discernment**

Age

CRT

no-conflict

MTD

Path c = .006***

Path a = .52***

Path b = .0004*

Direct effect: 96.27% ***

Indirect effect: 3.73% *

Path c’= .0002*

Age

CRT

conflict

MTD

Path c = .004***

Path a = 2.50***

Path b = .0009***

Direct effect: 66.1% ***

Indirect effect: 33.9% ***

Path c’= .002***

**(B) fake news**

Age

CRT

conflict

Fake news

Path c = -.0003

Path a = 2.50***

Path b = -.002***

Direct effect: 4.86%

Indirect effect: 95.14%***

Path c’= -.005***

Age

CRT

no-conflict

Fake news

Path c = -.005*

Path a = .52***

Path b = -.001

Direct effect: 90.72% *

Indirect effect: 9.28%

Path c’= -.0005

**(C) real news**

Age

CRT

conflict

Real news

Path c = .01***

Path a = 2.50***

Path b = .0003

Direct effect: 92.92% ***

Indirect effect: 7.08%

Path c’= .001

Age

CRT

no-conflict

Real news

Path c = .01***

Path a = .52***

Path b = .0003

Direct effect: 98.71% ***

Indirect effect: 1.29%

Path c’= 0.0001

Fig. S4. Development of the ability to evaluate the veracity of news from young adolescents to adults, restricted to repeated news items. (A) Mean accuracy ratings concerning real and fake news items as a function of age group (scored using a Likert scale ranging from 1: “not accurate at all” to 4: “very accurate”). (B) Distribution of the mean accuracy ratings pertaining to real and fake new items for each age group. (C) Mean media truth discernment scores (higher scores reflect greater accuracy with regard to discriminating between real and fake news) as a function of age group. (D) Distribution of the mean media truth discernment scores for each age group. Error bars indicate 95% confidence intervals.


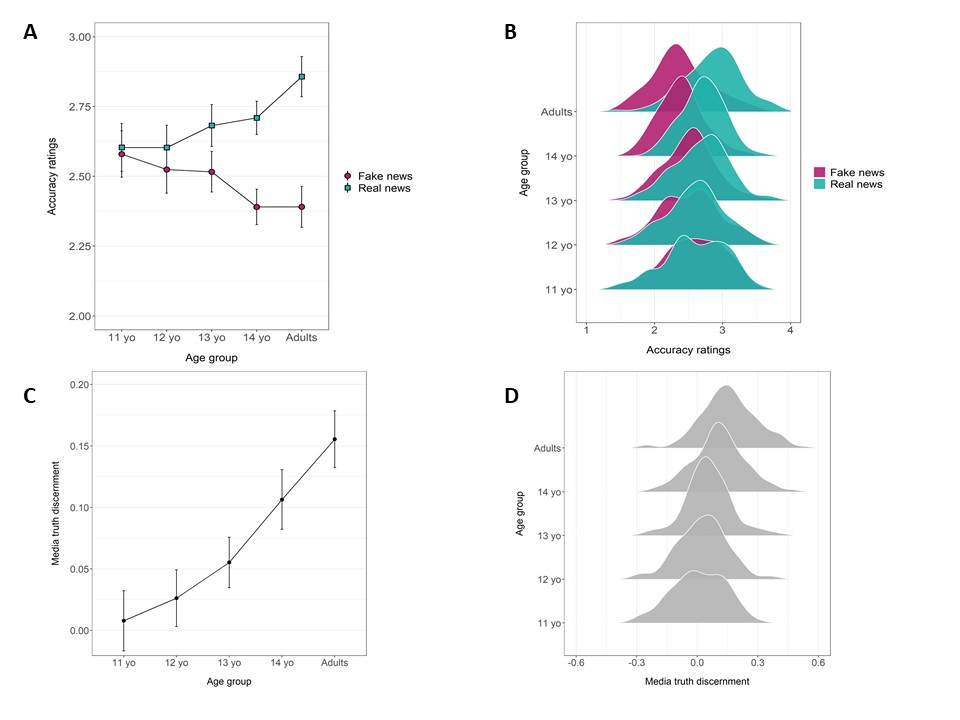


Fig. S5. Mediation analysis restricted to repeated news items, including CRT conflict and no-conflict scores as mediators, age as the independent variable and (A) media truth discernment, (B) fake news items and (C) real news items as dependent variables. Path c reflects the direct effect, while path c’ reflect the indirect effect. The percentage of mediation indicates the percentage of the total effect for which the indirect and direct effects account (using the “medmod” module in Jamovi 2.3.24 software). Note. * p < .05, ** p < .01, *** p < .001

**(A) media truth discernment**

Age

CRT

conflict

Fake news

Path c = -.003

Path a = 2.50***

Path b = -.001*

Direct effect: 51.2%

Indirect effect: 48.8%*

Path c’= -.003*

Age

CRT

no-conflict

Fake news

Path c = -.006*

Path a = .52***

Path b = -.0001

Direct effect: 98.95% *

Indirect effect: 1.05%

Path c’= -.00007

Age

CRT

no-conflict

MTD

Path c = .006***

Path a = .52***

Path b = .0004

Direct effect: 96.93% ***

Indirect effect: 3.07%

Path c’= .0002

Age

CRT

conflict

MTD

Path c = .005***

Path a = 2.50***

Path b = .0006***

Direct effect: 75.0% ***

Indirect effect: 25.0% ***

Path c’= .001***

**(B) fake news**

Age

CRT

conflict

Real news

Path c = .01***

Path a = 2.50***

Path b = .0007

Direct effect: 87.0% ***

Indirect effect: 13.0%

Path c’= .001

Age

CRT

no-conflict

Real news

Path c = .01***

Path a = .52***

Path b = .001

Direct effect: 95.92% ***

Indirect effect: 4.08%

Path c’= 0.0005

**(C) real news**

Table S1. Summary statistics of media truth discernment scores per age group.

SD stands for standard deviations, 95% CI for 95% confidence interval, min-max for the minimum and maximum scores.

|  |  | mtd |
| --- | --- | --- |
| 11 years old | *M* (*SD*) | 0.016 (0.09) |
|  | 95% CI | [-0.001 ; 0.03] |
|  | min-max | -0.20 ; 0.29 |
|  | skewness | 0.32 |
| 12 years old | *M* (*SD*) | 0.041 (0.09) |
|  | 95% CI | [0.02 ; 0.06] |
|  | min-max | -0.21 ; 0.35 |
|  | skewness | 0.05 |
| 13 years old | *M* (*SD*) | 0.077 (0.08) |
|  | 95% CI | [0.06 ; 0.09] |
|  | min-max | -0.10 ; 0.29 |
|  | skewness | 0.17 |
| 14 years old | *M* (*SD*) | 0.103 (0.09) |
|  | 95% CI | [0.08 ; 0.12] |
|  | min-max | -0.19 ; 0.36 |
|  | skewness | -0.19 |
| Adults | *M* (*SD*) | 0.166 (0.13) |
|  | 95% CI | [0.14 ; 0.19] |
|  | min-max | -0.20 ; 0.51 |
|  | skewness | -0.08 |

Table S2. Summary statistics of accuracy ratings for real and fake news items per age group.

*SD* stands for standard deviations, 95% CI for 95% confidence interval, min-max for the minimum and maximum scores.

|  |  | Real news | Fake news |
| --- | --- | --- | --- |
| 11 years old | *M* (*SD*) | 2.55 (0.37) | 2.50 (0.37) |
|  | 95% CI | [2.48 ; 2.62] | [2.43 ; 2.57] |
|  | min-max | 1.57 - 3.47 | 1.61 - 3.25 |
|  | skewness | - 0.25 | -0.29 |
| 12 years old | *M* (*SD*) | 2.57 (0.38) | 2.44 (0.37) |
|  | 95% CI | [2.49 ; 2.64] | [2.37 ; 2.51] |
|  | min-max | 1.68 - 3.47 | 1.47 - 3.29 |
|  | skewness | -0.28 | 0.04 |
| 13 years old | *M* (*SD*) | 2.62 (0.33) | 2.39 (0.32) |
|  | 95% CI | [2.56 ; 2.68] | [2.33 ; 2.45] |
|  | min-max | 1.76 - 3.29 | 1.69 - 3.29 |
|  | skewness | -0.35 | 0.15 |
| 14 years old | *M* (*SD*) | 2.60 (0.28) | 2.29 (0.28) |
|  | 95% CI | [2.55 ; 2.66] | [2.24 ; 2.35] |
|  | min-max | 2.00 - 3.36 | 1.54 - 3.01 |
|  | skewness | - 0.16 | 0.32 |
| Adults | *M* (*SD*) | 2.80 (0.36) | 2.31 (0.37) |
|  | 95% CI | [2.74 ; 2.87] | [2.24 ; 2.37] |
|  | min-max | 1.58 - 3.64 | 1.43 - 3.24 |
|  | skewness | - 0.17 | 0.45 |

Table S3. Developmental effect of the media truth discernment scores.

| (a) ANOVA table | | | | | | | | | | | | | |
| --- | --- | --- | --- | --- | --- | --- | --- | --- | --- | --- | --- | --- | --- |
|  | | Sum of Squares | | *df* | | Mean Square | | *F* | | *p* | | *η²_p_* [95% CI] | |
| Age group |  | 1.64 |  | 4 |  | 0.4095 |  | 38.5 |  | < .001 |  | 0.22 [0.17, 1.00] |  |
| Residuals |  | 5.94 |  | 559 |  | 0.0106 |  |  |  |  |  |  |  |
|  | | | | | | | | | | | | | |

(b) Polynomial contrasts

|  | Estimate | se | t | p |
| --- | --- | --- | --- | --- |
| linear | 0.11439 | 0.00953 | 12.004 | < .001 |
| quadratic | 0.01765 | 0.00963 | 1.833 | 0.067 |
| cubic | 0.00852 | 0.00988 | 0.863 | 0.389 |
| quartic | 0.00830 | 0.00991 | 0.837 | 0.403 |

(c) Post-hoc analysis with holm correction

| Comparisons | Mean difference | se | df | t | p _holm_ | Cohen's d | 95% CI |
| --- | --- | --- | --- | --- | --- | --- | --- |
| 11 yo - 12 yo | -0.0251 | 0.0140 | 559 | -1.80 | 0.14 | -0.15 | [-0.32; 0.01] |
| 12 yo - 13 yo | -0.0360 | 0.0140 | 559 | -2.57 | 0.03 | -0.22 | [-0.38; -0.05] |
| 13 yo – 14 yo | -0.0256 | 0.0141 | 559 | -1.82 | 0.14 | -0.15 | [-0.32; 0.01] |
| 14 yo - adults | -0.0634 | 0.0135 | 559 | -4.70 | < .001 | -0.40 | [-0.56; -0.23] |

Table S4. Developmental effect of mean accuracy ratings for real and fake news items.

(a) ANOVA table

| Within Subjects Effects | | | | | | | | | | | | |  |
| --- | --- | --- | --- | --- | --- | --- | --- | --- | --- | --- | --- | --- | --- |
|  | | Sum of Squares | | *df* | | Mean Square | | *F* | | *p* | | *η²_p_* [95% CI] |  |
| Veracity |  | 33.054 |  | 1 |  | 33.0543 |  | 345.35 |  | < .001 |  | 0.38 [0.33, 1.00] |  |
| Veracity ✻ Age group |  | 14.712 |  | 4 |  | 3.6781 |  | 38.43 |  | < .001 |  | 0.22 [0.16, 1.00] |  |
| Residual |  | 53.504 |  | 559 |  | 0.0957 |  |  |  |  |  |  |  |
| Repetition |  | 14.542 |  | 1 |  | 14.5421 |  | 164.78 |  | < .001 |  | 0.23 [0.18, 1.00] |  |
| Repetition ✻ Age group |  | 0.644 |  | 4 |  | 0.1610 |  | 1.82 |  | 0.123 |  | 0.01 [0.00, 1.00] |  |
| Residual |  | 49.333 |  | 559 |  | 0.0883 |  |  |  |  |  |  |  |
| Veracity ✻ Repetition |  | 0.582 |  | 1 |  | 0.5819 |  | 11.08 |  | < .001 |  | 0.02 [0.00, 1.00] |  |
| Veracity ✻ Repetition ✻ Age group |  | 0.341 |  | 4 |  | 0.0851 |  | 1.62 |  | 0.167 |  | 0.01 [0.00, 1.00] |  |
| Residual |  | 29.358 |  | 559 |  | 0.0525 |  |  |  |  |  |  |  |
| \| Between Subjects Effects \| \| \| \| \| \| \| \| \| \| \| \| \| \| --- \| --- \| --- \| --- \| --- \| --- \| --- \| --- \| --- \| --- \| --- \| --- \| --- \| \|  \| \| Sum of Squares \| \| *df* \| \| Mean Square \| \| *F* \| \| *p* \| \| *η²_p_* [95% CI] \| \| Age group \|  \| 2.93 \|  \| 4 \|  \| 0.733 \|  \| 1.89 \|  \| 0.111 \|  \| 0.01 [0.00, 1.00] \|  \| \| Residual \|  \| 216.85 \|  \| 559 \|  \| 0.388 \|  \|  \|  \|  \|  \|  \|  \| \| Note. Type 3 Sums of Squares \| \| \| \| \| \| \| \| \| \| \| \| \| \|  \| \| \| \| \| \| \| \| \| \| \| \| \| | | | | | | | | | | | | |  |

(b) Post-hoc analysis with holm correction

| Age group | real news items (mean accuracy ratings) | fake news items (mean accuracy ratings) | t (df) | p _holm_ | Cohen's d | 95% CI |
| --- | --- | --- | --- | --- | --- | --- |
| 11 yo | 2.55 (0.37) | 2.50 (0.37) | 1.66 (559) | 0.09 | 0.07 | [-0.01; 0.15] |
| 12 yo | 2.57 (0.38) | 2.44 (0.36) | 4.19 (559) | <.001 | 0.18 | [0.09; 0.26] |
| 13 yo | 2.62 (0.33) | 2.39 (0.32) | 7.83 (559) | <.001 | 0.33 | [0.25; 0.42] |
| 14 yo | 2.60 (0.28) | 2.29 (0.28) | 10.24 (559) | <.001 | 0.43 | [0.35; 0.52] |
| Adults | 2.80 (0.36) | 2.31 (0.36) | 18.53 (559) | <.001 | 0.78 | [0.69; 0.88] |

|  |  | mtd | real news | fake news | ite | crt conflict | crt no-conflict |
| --- | --- | --- | --- | --- | --- | --- | --- |
| mtd | Pearson's *r* | — |  |  |  |  |  |
|  | *p*-value | — |  |  |  |  |  |
| real news | Pearson's *r* | 0.452*** | — |  |  |  |  |
|  | *p*-value | < .001 | — |  |  |  |  |
| fake news | Pearson's *r* | -0.462*** | 0.583*** | — |  |  |  |
|  | *p*-value | < .001 | < .001 | — |  |  |  |
| ite | Pearson's *r* | 0.033 | 0.131* | 0.101* | — |  |  |
|  | *p*-value | 0.437 | 0.002 | 0.017 | — |  |  |
| crt conflict | Pearson's *r* | 0.236*** | 0.050 | -0.165*** | 0.070 | — |  |
|  | *p*-value | < .001 | 0.238 | < .001 | 0.095 | — |  |
| crt no- conflict | Pearson's *r* | 0.106* | 0.052 | -0.045 | 0.072 | 0.156*** | — |
|  | *p*-value | 0.014 | 0.217 | 0.288 | 0.089 | < .001 | — |

Table S5. Partial Pearson correlations controlled for age.

| Note. * *p* < .05, ** *p* < .01, *** *p* < .001 |
| --- |

Table S6. Summary statistics of accuracy rate (%) for CRT conflict and no-conflict items in each age group.

Means are in % and standard deviation are in parentheses.

|  | crt  conflict items | crt  no-conflict items |
| --- | --- | --- |
| 11 years old | 8.18 (19.8) | 80.9 (28.7) |
| 12 years old | 9.72 (21.0) | 84.3 (25.3) |
| 13 years old | 15.1 (29.3) | 83.5 (28.1) |
| 14 years old | 22.9 (32.5) | 84.3 (25.3) |
| Adults | 56.4 (41.2) | 91.3 (20.0) |

Table S7. Results of mediation analysis with age as the independent variable, media truth discernment as the dependent variable, and CRT conflict (A) and CRT no-conflict (B) as mediators.

| (A) CRT conflict items as mediator  Mediation Estimates | | | | | | | | | | | | | | | | | |
| --- | --- | --- | --- | --- | --- | --- | --- | --- | --- | --- | --- | --- | --- | --- | --- | --- | --- |
|  | | | | | | | | **95% Confidence Interval** | | | |  | | | | | |
| **Effect** | | **Label** | | **Estimate** | | ***SE*** | | **Lower** | | **Upper** | | ***Z*** | | ***p*** | | **% Mediation** | |
| Indirect |  | a × b |  | 0.00201 |  | 4.23e-4 |  | 0.00121 |  | 0.00298 |  | 4.74 |  | < .001 |  | 29.4 |  |
| Direct |  | c |  | 0.00482 |  | 8.36e-4 |  | 0.00322 |  | 0.00647 |  | 5.76 |  | < .001 |  | 70.6 |  |
| Total |  | c + a × b |  | 0.00683 |  | 8.32e-4 |  | 0.00526 |  | 0.00852 |  | 8.20 |  | < .001 |  | 100.0 |  |
|  | | | | | | | | | | | | | | | | | |

| Path Estimates | | | | | | | | | | | | | | | | | | | |
| --- | --- | --- | --- | --- | --- | --- | --- | --- | --- | --- | --- | --- | --- | --- | --- | --- | --- | --- | --- |
|  | | | | | | | | | | | | **95% Confidence Interval** | | | |  | | | |
|  | |  | |  | | **Label** | | **Estimate** | | ***SE*** | | **Lower** | | **Upper** | | ***Z*** | | ***p*** | |
| age |  | → |  | crt_conflict |  | a |  | 2.50402 |  | 0.265 |  | 2.01846 |  | 3.05302 |  | 9.44 |  | < .001 |  |
| crt_conflict |  | → |  | mtd |  | b |  | 8.02e-4 |  | 1.45e-4 |  | 5.20e-4 |  | 0.00109 |  | 5.51 |  | < .001 |  |
| age |  | → |  | mtd |  | c |  | 0.00482 |  | 8.36e-4 |  | 0.00322 |  | 0.00647 |  | 5.76 |  | < .001 |  |
|  | | | | | | | | | | | | | | | | | | | |

(B) CRT no-conflict as mediator

| Mediation Estimates | | | | | | | | | | | | | | | | | |
| --- | --- | --- | --- | --- | --- | --- | --- | --- | --- | --- | --- | --- | --- | --- | --- | --- | --- |
|  | | | | | | | | **95% Confidence Interval** | | | |  | | | | | |
| **Effect** | | **Label** | | **Estimate** | | ***SE*** | | **Lower** | | **Upper** | | ***Z*** | | ***p*** | | **% Mediation** | |
| Indirect |  | a × b |  | 2.33e-4 |  | 1.03e-4 |  | 4.71e-5 |  | 4.56e-4 |  | 2.27 |  | 0.023 |  | 3.42 |  |
| Direct |  | c |  | 0.00659 |  | 8.01e-4 |  | 0.00501 |  | 0.00810 |  | 8.23 |  | < .001 |  | 96.58 |  |
| Total |  | c + a × b |  | 0.00683 |  | 8.11e-4 |  | 0.00520 |  | 0.00839 |  | 8.41 |  | < .001 |  | 100.00 |  |
|  | | | | | | | | | | | | | | | | | |

| Path Estimates | | | | | | | | | | | | | | | | | | | |
| --- | --- | --- | --- | --- | --- | --- | --- | --- | --- | --- | --- | --- | --- | --- | --- | --- | --- | --- | --- |
|  | | | | | | | | | | | | **95% Confidence Interval** | | | |  | | | |
|  | |  | |  | | **Label** | | **Estimate** | | ***SE*** | | **Lower** | | **Upper** | | ***Z*** | | ***p*** | |
| age |  | → |  | crt_no-conflict |  | a |  | 0.52358 |  | 0.133 |  | 0.27570 |  | 0.79745 |  | 3.94 |  | < .001 |  |
| crt_no-conflict |  | → |  | mtd |  | b |  | 4.46e-4 |  | 1.70e-4 |  | 9.45e-5 |  | 7.54e-4 |  | 2.63 |  | 0.009 |  |
| age |  | → |  | mtd |  | c |  | 0.00659 |  | 8.01e-4 |  | 0.00501 |  | 0.00810 |  | 8.23 |  | < .001 |  |
|  | | | | | | | | | | | | | | | | | | | |

Table S8. Results of mediation analysis with age as the independent variable, fake news accuracy ratings as the dependent variable, and CRT conflict (A) and CRT no-conflict (B) as mediators.

(A) CRT conflict items as mediator

| Mediation Estimates | | | | | | | | | | | | | | | | | |
| --- | --- | --- | --- | --- | --- | --- | --- | --- | --- | --- | --- | --- | --- | --- | --- | --- | --- |
|  | | | | | | | | **95% Confidence Interval** | | | |  | | | | | |
| **Effect** | | **Label** | | **Estimate** | | **SE** | | **Lower** | | **Upper** | | **Z** | | **p** | | **% Mediation** | |
| Indirect |  | a × b |  | -0.00463 |  | 0.00123 |  | -0.00731 |  | -0.00240 |  | -3.776 |  | < .001 |  | 69.7 |  |
| Direct |  | c |  | -0.00201 |  | 0.00241 |  | -0.00693 |  | 0.00283 |  | -0.835 |  | 0.404 |  | 30.3 |  |
| Total |  | c + a × b |  | -0.00664 |  | 0.00224 |  | -0.01119 |  | -0.00220 |  | -2.963 |  | 0.003 |  | 100.0 |  |
|  | | | | | | | | | | | | | | | | | |

| Path Estimates | | | | | | | | | | | | | | | | | | | |
| --- | --- | --- | --- | --- | --- | --- | --- | --- | --- | --- | --- | --- | --- | --- | --- | --- | --- | --- | --- |
|  | | | | | | | | | | | | **95% Confidence Interval** | | | |  | | | |
|  | |  | |  | | **Label** | | **Estimate** | | **SE** | | **Lower** | | **Upper** | | **Z** | | **p** | |
| age |  | → |  | crt_conflict |  | a |  | 2.50402 |  | 0.26771 |  | 1.97813 |  | 3.02206 |  | 9.353 |  | < .001 |  |
| crt_conflict |  | → |  | mean_fake |  | b |  | -0.00185 |  | 4.53e-4 |  | -0.00274 |  | -9.62e−4 |  | -4.082 |  | < .001 |  |
| age |  | → |  | mean_fake |  | c |  | -0.00201 |  | 0.00241 |  | -0.00693 |  | 0.00283 |  | -0.835 |  | 0.404 |  |
|  | | | | | | | | | | | | | | | | | | | |

(B) CRT no-conflict as mediator

| Mediation Estimates | | | | | | | | | | | | | | | | | |
| --- | --- | --- | --- | --- | --- | --- | --- | --- | --- | --- | --- | --- | --- | --- | --- | --- | --- |
|  | | | | | | | | **95% Confidence Interval** | | | |  | | | | | |
| **Effect** | | **Label** | | **Estimate** | | **SE** | | **Lower** | | **Upper** | | **Z** | | **p** | | **% Mediation** | |
| Indirect |  | a × b |  | -3.25e−4 |  | 3.19e-4 |  | -9.94e−4 |  | 3.67e-4 |  | -1.02 |  | 0.308 |  | 4.89 |  |
| Direct |  | c |  | -0.00632 |  | 0.00228 |  | -0.0107 |  | -0.00193 |  | -2.77 |  | 0.006 |  | 95.11 |  |
| Total |  | c + a × b |  | -0.00664 |  | 0.00224 |  | -0.0110 |  | -0.00223 |  | -2.97 |  | 0.003 |  | 100.00 |  |
|  | | | | | | | | | | | | | | | | | |

| Path Estimates | | | | | | | | | | | | | | | | | | | |
| --- | --- | --- | --- | --- | --- | --- | --- | --- | --- | --- | --- | --- | --- | --- | --- | --- | --- | --- | --- |
|  | | | | | | | | | | | | **95% Confidence Interval** | | | |  | | | |
|  | |  | |  | | **Label** | | **Estimate** | | **SE** | | **Lower** | | **Upper** | | **Z** | | **p** | |
| age |  | → |  | crt_no-conflict |  | a |  | 0.52358 |  | 0.13118 |  | 0.26572 |  | 0.77980 |  | 3.99 |  | < .001 |  |
| crt_no-conflict |  | → |  | mean_fake |  | b |  | -6.20e−4 |  | 6.01e-4 |  | -0.00178 |  | 5.91e-4 |  | -1.03 |  | 0.302 |  |
| age |  | → |  | mean_fake |  | c |  | -0.00632 |  | 0.00228 |  | -0.01073 |  | -0.00193 |  | -2.77 |  | 0.006 |  |
|  | | | | | | | | | | | | | | | | | | | |

Table S9. Results of mediation analysis with age as the independent variable, real news accuracy ratings as the dependent variable, and CRT conflict (A) and CRT no-conflict (B) as mediators.

(A) CRT conflict items as mediator

| Mediation Estimates | | | | | | | | | | | | | | | | | |
| --- | --- | --- | --- | --- | --- | --- | --- | --- | --- | --- | --- | --- | --- | --- | --- | --- | --- |
|  | | | | | | | | **95% Confidence Interval** | | | |  | | | | | |
| **Effect** | | **Label** | | **Estimate** | | **SE** | | **Lower** | | **Upper** | | **Z** | | **p** | | **% Mediation** | |
| Indirect |  | a × b |  | 0.00139 |  | 0.00109 |  | -7.74e−4 |  | 0.00364 |  | 1.27 |  | 0.204 |  | 10.0 |  |
| Direct |  | c |  | 0.01245 |  | 0.00265 |  | 0.00739 |  | 0.01757 |  | 4.70 |  | < .001 |  | 90.0 |  |
| Total |  | c + a × b |  | 0.01383 |  | 0.00235 |  | 0.00944 |  | 0.01856 |  | 5.89 |  | < .001 |  | 100.0 |  |
|  | | | | | | | | | | | | | | | | | |

| Path Estimates | | | | | | | | | | | | | | | | | | | |
| --- | --- | --- | --- | --- | --- | --- | --- | --- | --- | --- | --- | --- | --- | --- | --- | --- | --- | --- | --- |
|  | | | | | | | | | | | | **95% Confidence Interval** | | | |  | | | |
|  | |  | |  | | **Label** | | **Estimate** | | **SE** | | **Lower** | | **Upper** | | **Z** | | **p** | |
| age |  | → |  | crt_conflict |  | a |  | 2.5040 |  | 0.26484 |  | 1.98698 |  | 3.02437 |  | 9.45 |  | < .001 |  |
| crt_conflict |  | → |  | mean_real |  | b |  | 5.54e-4 |  | 4.26e-4 |  | -3.51e−4 |  | 0.00139 |  | 1.30 |  | 0.194 |  |
| age |  | → |  | mean_real |  | c |  | 0.0124 |  | 0.00265 |  | 0.00739 |  | 0.01757 |  | 4.70 |  | < .001 |  |
|  | | | | | | | | | | | | | | | | | | | |

(B) CRT no-conflict as mediator

| Mediation Estimates | | | | | | | | | | | | | | | | | |
| --- | --- | --- | --- | --- | --- | --- | --- | --- | --- | --- | --- | --- | --- | --- | --- | --- | --- |
|  | | | | | | | | **95% Confidence Interval** | | | |  | | | | | |
| **Effect** | | **Label** | | **Estimate** | | **SE** | | **Lower** | | **Upper** | | **Z** | | **p** | | **% Mediation** | |
| Indirect |  | a × b |  | 3.75e-4 |  | 3.39e-4 |  | -2.76e−4 |  | 0.00109 |  | 1.11 |  | 0.268 |  | 2.71 |  |
| Direct |  | c |  | 0.0135 |  | 0.00239 |  | 0.00874 |  | 0.01851 |  | 5.63 |  | < .001 |  | 97.29 |  |
| Total |  | c + a × b |  | 0.0138 |  | 0.00236 |  | 0.00906 |  | 0.01889 |  | 5.87 |  | < .001 |  | 100.00 |  |
|  | | | | | | | | | | | | | | | | | |

| Path Estimates | | | | | | | | | | | | | | | | | | | |
| --- | --- | --- | --- | --- | --- | --- | --- | --- | --- | --- | --- | --- | --- | --- | --- | --- | --- | --- | --- |
|  | | | | | | | | | | | | **95% Confidence Interval** | | | |  | | | |
|  | |  | |  | | **Label** | | **Estimate** | | **SE** | | **Lower** | | **Upper** | | **Z** | | **p** | |
| age |  | → |  | crt_no-conflict |  | a |  | 0.5236 |  | 0.13243 |  | 0.26264 |  | 0.78617 |  | 3.95 |  | < .001 |  |
| crt_no-conflict |  | → |  | mean_real |  | b |  | 7.17e-4 |  | 5.92e-4 |  | -4.90e−4 |  | 0.00190 |  | 1.21 |  | 0.226 |  |
| age |  | → |  | mean_real |  | c |  | 0.0135 |  | 0.00239 |  | 0.00874 |  | 0.01851 |  | 5.63 |  | < .001 |  |
|  | | | | | | | | | | | | | | | | | | | |

Table S10. Summary statistics of media truth discernment scores per age group, restricted to novel news items.

SD stands for standard deviations, 95% CI for 95% confidence interval, min-max for the minimum and maximum scores.

|  |  | mtd |
| --- | --- | --- |
| 11 years old | *M* (*SD*) | 0.02 (0.12) |
|  | 95% CI | [0.002 ; 0.04] |
|  | min-max | -0.22 ; 0.33 |
|  | skewness | 0.265 |
| 12 years old | *M* (*SD*) | 0.05 (0.12) |
|  | 95% CI | [0.03 ; 0.08] |
|  | min-max | -0.28 ; 0.38 |
|  | skewness | 0.191 |
| 13 years old | *M* (*SD*) | 0.09 (0.11) |
|  | 95% CI | [0.07 ; 0.12] |
|  | min-max | -0.13 ; 0.45 |
|  | skewness | 0.467 |
| 14 years old | *M* (*SD*) | 0.09 (0.11) |
|  | 95% CI | [0.07; 0.12] |
|  | min-max | -0.25 ; 0.35 |
|  | skewness | -0.312 |
| Adults | *M* (*SD*) | 0.17 (0.16) |
|  | 95% CI | [0.15 ; 0.20] |
|  | min-max | -0.23 ; 0.63 |
|  | skewness | 0.027 |

Table S11. Summary statistics of mean accuracy ratings per age group restricted to novel news items.

SD stands for standard deviations, 95% CI for 95% confidence interval, min-max for the minimum and maximum scores.

|  |  | Real news | Fake news |
| --- | --- | --- | --- |
| 11 years old | *M* (*SD*) | 2.49 (0.376) | 2.42 (0.394) |
|  | 95% CI | [2.42 ; 2.56] | [2.34 ; 2.49] |
|  | min-max | 1.57 ; 3.43 | 1.36 ; 3.29 |
|  | skewness | 0.08 | -0.261 |
| 12 years old | *M* (*SD*) | 2.52 (0.415) | 2.35 (0.392) |
|  | 95% CI | [2.44 ; 2.60] | [2.28 ; 2.43] |
|  | min-max | 1.67 ; 3.36 | 1.43 ; 3.14 |
|  | skewness | -0.252 | 0.014 |
| 13 years old | *M* (*SD*) | 2.55 (0.378) | 2.25 (0.372) |
|  | 95% CI | [2.48 ; 2.62] | [2.18 ; 2.32] |
|  | min-max | 1.67 ; 3.43 | 1.50 ; 3.14 |
|  | skewness | - 0.159 | 0.282 |
| 14 years old | *M* (*SD*) | 2.49 (0.347) | 2.19 (0.342) |
|  | 95% CI | [2.42 ; 2.56] | [2.13 ; 2.26] |
|  | min-max | 1.63 ; 3.43 | 1.21 ; 3.27 |
|  | skewness | 0.150 | 0.168 |
| Adults | *M* (*SD*) | 2.75 (0.398) | 2.22 (0.377) |
|  | 95% CI | [2.68 ; 2.82] | [2.15 ; 2.28] |
|  | min-max | 1.75 ; 3.86 | 1.20 ; 3.27 |
|  | skewness | -0.372 | 0.367 |

Table S12. Developmental effect on the media truth discernment, restricted to novel news items

| (A) ANOVA table | | | | | | | | | | | | | | |
| --- | --- | --- | --- | --- | --- | --- | --- | --- | --- | --- | --- | --- | --- | --- |
|  | | Sum of Squares | | *df* | | Mean Square | | *F* | | *p* | | *η²_p_* [95% CI] | | |
| Age group |  | 1.59 |  | 4 |  | 0.3983 |  | 23.2 |  | < .001 |  | 0.142 [0.10; 1.00] |  |  |
| Residuals |  | 9.59 |  | 559 |  | 0.0172 |  |  |  |  |  |  |  |  |
|  | | | | | | | | | | | | | |  |

(B) Polynomial contrasts

|  | Estimate | se | t | p |
| --- | --- | --- | --- | --- |
| linear | 0.1099 | 0.0121 | 9.07 | < .001 |
| quadratic | 0.0129 | 0.0122 | 1.05 | 0.292 |
| cubic | 0.0211 | 0.0125 | 1.68 | 0.093 |
| quartic | 0.0206 | 0.0126 | 1.63 | 0.103 |

(C) Post-hoc analysis with holm correction

| Comparisons | Mean difference | se | df | t | p _holm_ | Cohen's d | 95% CI |
| --- | --- | --- | --- | --- | --- | --- | --- |
| 11 yo - 12 yo | -0.0321 | 0.0177 | 559 | -1.8107 | 0.142 | -0.15 | [-0.32; 0.01] |
| 12 yo - 13 yo | -0.0425 | 0.0178 | 559 | -2.3918 | 0.051 | -0.20 | [-0.37; -0.04] |
| 13 yo – 14 yo | -2.38e−4 | 0.0179 | 559 | -0.0133 | 0.989 | -0.00 | [-0.17; 0.16] |
| 14 yo - adults | -0.0774 | 0.0171 | 559 | -4.5196 | < .001 | -0.38 | [-0.55; -0.21] |

Table S13. Developmental effect of mean accuracy ratings for real and fake news items, restricted to novel news items

(A) ANOVA table

 Within Subjects effects

|  | | Sum of Squares | | *df* | | Mean Square | | *F* | | *p* | | *η²_p_* [95% CI] |  |
| --- | --- | --- | --- | --- | --- | --- | --- | --- | --- | --- | --- | --- | --- |
| Veracity |  | 21.20 |  | 1 |  | 21.2039 |  | 274.6 |  | < .001 |  | 0.329 [0.28; 1.00] |  |
| Veracity ✻ Age group |  | 7.17 |  | 4 |  | 1.7923 |  | 23.5 |  | < .001 |  | 0.142 [0.10; 1.00] |  |
| Residual |  | 43.16 |  | 559 |  | 0.0772 |  |  |  |  |  |  |  |
|  | | | | | | | | | | | | |  |
|  | | | | | | | | | | | | |  |

| Between Subjects Effects | | | | | | | | | | | | | |
| --- | --- | --- | --- | --- | --- | --- | --- | --- | --- | --- | --- | --- | --- |
|  | | Sum of Squares | | *df* | | Mean Square | | *F* | | *p* | | *η²_p_* [95% CI] | |
| Age group |  | 2.77 |  | 4 |  | 0.694 |  | 3.27 |  | 0.011 |  | 0.023 [0.00; 1.00] |  |
| Residual |  | 118.50 |  | 559 |  | 0.212 |  |  |  |  |  |  |  |
| Note. Type 3 Sums of Squares | | | | | | | | | | | | | |
|  | | | | | | | | | | | | | |

(B) Post-hoc analysis with holm correction

| Age group | real news items (mean accuracy ratings) | fake news items (mean accuracy ratings) | t(df) | p_holm_ | Cohen's d | 95% CI |
| --- | --- | --- | --- | --- | --- | --- |
| 11 yo | 2.49 (0.37) | 2.42 (0.39) | 1.99 (559) | 0.05 | 0.08 | [0.00; 0.17] |
| 12 yo | 2.52 (0.41) | 2.35 (0.39) | 4.52 (559) | <.001 | 0.19 | [0.11; 0.27] |
| 13 yo | 2.55 (0.37) | 2.25 (0.37) | 7.93 (559) | <.001 | 0.34 | [0.25; 0.42] |
| 14 yo | 2.49 (0.34) | 2.19 (0.34) | 7.80 (559) | <.001 | 0.33 | [0.24; 0.42] |
| Adults | 2.75 (0.39) | 2.22 (0.37) | 15.54 (559) | <.001 | 0.66 | [0.57; 0.75] |

Table S14. Partial Pearson correlations controlled for age, restricted to novel news items.

|  |  | mtd | real news | fake news | crt conflict | crt no-conflict |
| --- | --- | --- | --- | --- | --- | --- |
| mtd | Pearson's *r* | — |  |  |  |  |
|  | *p*-value | — |  |  |  |  |
| real news | Pearson's *r* | 0.527*** | — |  |  |  |
|  | *p*-value | < .001 | — |  |  |  |
| fake news | Pearson's *r* | -0.517*** | 0.455*** | — |  |  |
|  | *p*-value | < .001 | < .001 | — |  |  |
| crt conflict | Pearson's *r* | 0.215*** | 0.033 | -0.193*** | — |  |
|  | *p*-value | < .001 | 0.441 | < .001 | — |  |
| crt non conflict | Pearson's *r* | 0.089* | 0.023 | -0.073 | 0.156*** | — |
|  | *p*-value | 0.034 | 0.586 | 0.084 | < .001 | — |

Note. * *p* < .05, ** *p* < .01, *** *p* < .001

Table S15. Results of mediation analysis with age as the independent variable, media truth discernment as the dependent variable, and CRT conflict (A) and CRT no-conflict (B) as mediators, restricted to novel news items.

(A) CRT conflict items as mediator

| Mediation Estimates | | | | | | | | | | | | | | | | | |
| --- | --- | --- | --- | --- | --- | --- | --- | --- | --- | --- | --- | --- | --- | --- | --- | --- | --- |
|  | | | | | | | | **95% Confidence Interval** | | | |  | | | | | |
| **Effect** | | **Label** | | **Estimate** | | ***SE*** | | **Lower** | | **Upper** | | ***Z*** | | ***p*** | | **% Mediation** | |
| Indirect |  | a × b |  | 0.00230 |  | 5.27e-4 |  | 0.00135 |  | 0.00340 |  | 4.36 |  | < .001 |  | 33.9 |  |
| Direct |  | c |  | 0.00448 |  | 9.87e-4 |  | 0.00256 |  | 0.00637 |  | 4.54 |  | < .001 |  | 66.1 |  |
| Total |  | c + a × b |  | 0.00677 |  | 9.86e-4 |  | 0.00480 |  | 0.00875 |  | 6.87 |  | < .001 |  | 100.0 |  |
|  | | | | | | | | | | | | | | | | | |

| Path Estimates | | | | | | | | | | | | | | | | | | | |
| --- | --- | --- | --- | --- | --- | --- | --- | --- | --- | --- | --- | --- | --- | --- | --- | --- | --- | --- | --- |
|  | | | | | | | | | | | | **95% Confidence Interval** | | | |  | | | |
|  | |  | |  | | **Label** | | **Estimate** | | ***SE*** | | **Lower** | | **Upper** | | ***Z*** | | ***p*** | |
| age |  | → |  | crt_conflict |  | a |  | 2.50402 |  | 0.271 |  | 1.99712 |  | 3.05494 |  | 9.25 |  | < .001 |  |
| crt_conflict |  | → |  | mdt_novel1 |  | b |  | 9.17e-4 |  | 1.82e-4 |  | 5.68e-4 |  | 0.00128 |  | 5.04 |  | < .001 |  |
| age |  | → |  | mdt_novel1 |  | c |  | 0.00448 |  | 9.87e-4 |  | 0.00256 |  | 0.00637 |  | 4.54 |  | < .001 |  |
|  | | | | | | | | | | | | | | | | | | | |

(B) CRT no-conflict items as mediator

| Mediation Estimates | | | | | | | | | | | | | | | | | |
| --- | --- | --- | --- | --- | --- | --- | --- | --- | --- | --- | --- | --- | --- | --- | --- | --- | --- |
|  | | | | | | | | **95% Confidence Interval** | | | |  | | | | | |
| **Effect** | | **Label** | | **Estimate** | | ***SE*** | | **Lower** | | **Upper** | | ***Z*** | | ***p*** | | **% Mediation** | |
| Indirect |  | a × b |  | 2.52e-4 |  | 1.20e-4 |  | 4.33e-5 |  | 5.29e-4 |  | 2.10 |  | 0.036 |  | 3.73 |  |
| Direct |  | c |  | 0.00652 |  | 9.87e-4 |  | 0.00441 |  | 0.00845 |  | 6.61 |  | < .001 |  | 96.27 |  |
| Total |  | c + a × b |  | 0.00677 |  | 9.91e-4 |  | 0.00473 |  | 0.00866 |  | 6.84 |  | < .001 |  | 100.00 |  |
|  | | | | | | | | | | | | | | | | | |

| Path Estimates | | | | | | | | | | | | | | | | | | | |
| --- | --- | --- | --- | --- | --- | --- | --- | --- | --- | --- | --- | --- | --- | --- | --- | --- | --- | --- | --- |
|  | | | | | | | | | | | | **95% Confidence Interval** | | | |  | | | |
|  | |  | |  | | **Label** | | **Estimate** | | ***SE*** | | **Lower** | | **Upper** | | ***Z*** | | ***p*** | |
| age |  | → |  | crt_no-conflict |  | a |  | 0.52358 |  | 0.133 |  | 0.25994 |  | 0.77863 |  | 3.93 |  | < .001 |  |
| crt_no-conflict |  | → |  | mdt_novel1 |  | b |  | 4.82e-4 |  | 2.06e-4 |  | 7.36e-5 |  | 9.02e-4 |  | 2.34 |  | 0.019 |  |
| age |  | → |  | mdt_novel1 |  | c |  | 0.00652 |  | 9.87e-4 |  | 0.00441 |  | 0.00845 |  | 6.61 |  | < .001 |  |
|  | | | | | | | | | | | | | | | | | | | |

Table S16. Results of mediation analysis with age as the independent variable, fake news accuracy ratings as the dependent variable, and CRT conflict (A) and CRT no-conflict (B) as mediators, restricted to novel news items.

(A) CRT conflict items as mediator

| Mediation Estimates | | | | | | | | | | | | | | | | | |
| --- | --- | --- | --- | --- | --- | --- | --- | --- | --- | --- | --- | --- | --- | --- | --- | --- | --- |
|  | | | | | | | | **95% Confidence Interval** | | | |  | | | | | |
| **Effect** | | **Label** | | **Estimate** | | ***SE*** | | **Lower** | | **Upper** | | ***Z*** | | ***p*** | | **% Mediation** | |
| Indirect |  | a × b |  | -0.00589 |  | 0.00134 |  | -0.00884 |  | -0.00337 |  | -4.401 |  | < .001 |  | 95.14 |  |
| Direct |  | c |  | -3.01e−4 |  | 0.00251 |  | -0.00501 |  | 0.00474 |  | -0.120 |  | 0.905 |  | 4.86 |  |
| Total |  | c + a × b |  | -0.00619 |  | 0.00229 |  | -0.01090 |  | -0.00175 |  | -2.706 |  | 0.007 |  | 100.00 |  |
|  | | | | | | | | | | | | | | | | | |

| Path Estimates | | | | | | | | | | | | | | | | | | | |
| --- | --- | --- | --- | --- | --- | --- | --- | --- | --- | --- | --- | --- | --- | --- | --- | --- | --- | --- | --- |
|  | | | | | | | | | | | | **95% Confidence Interval** | | | |  | | | |
|  | |  | |  | | **Label** | | **Estimate** | | ***SE*** | | **Lower** | | **Upper** | | ***Z*** | | ***p*** | |
| age |  | → |  | crt_conflict |  | a |  | 2.50402 |  | 0.26946 |  | 1.99094 |  | 3.05135 |  | 9.293 |  | < .001 |  |
| crt_conflict |  | → |  | fake_novel |  | b |  | -0.00235 |  | 4.83e-4 |  | -0.00332 |  | -0.00138 |  | -4.874 |  | < .001 |  |
| age |  | → |  | fake_novel |  | c |  | -3.01e−4 |  | 0.00251 |  | -0.00501 |  | 0.00474 |  | -0.120 |  | 0.905 |  |
|  | | | | | | | | | | | | | | | | | | | |

(B) CRT no-conflict items as mediator

| Mediation Estimates | | | | | | | | | | | | | | | | | |
| --- | --- | --- | --- | --- | --- | --- | --- | --- | --- | --- | --- | --- | --- | --- | --- | --- | --- |
|  | | | | | | | | **95% Confidence Interval** | | | |  | | | | | |
| **Effect** | | **Label** | | **Estimate** | | ***SE*** | | **Lower** | | **Upper** | | ***Z*** | | ***p*** | | **% Mediation** | |
| Indirect |  | a × b |  | -5.74e−4 |  | 3.48e-4 |  | -0.00126 |  | 4.03e-5 |  | -1.65 |  | 0.099 |  | 9.28 |  |
| Direct |  | c |  | -0.00562 |  | 0.00220 |  | -0.01001 |  | -0.00172 |  | -2.56 |  | 0.011 |  | 90.72 |  |
| Total |  | c + a × b |  | -0.00619 |  | 0.00221 |  | -0.01070 |  | -0.00233 |  | -2.80 |  | 0.005 |  | 100.00 |  |
|  | | | | | | | | | | | | | | | | | |

| Path Estimates | | | | | | | | | | | | | | | | | | | |
| --- | --- | --- | --- | --- | --- | --- | --- | --- | --- | --- | --- | --- | --- | --- | --- | --- | --- | --- | --- |
|  | | | | | | | | | | | | **95% Confidence Interval** | | | |  | | | |
|  | |  | |  | | **Label** | | **Estimate** | | ***SE*** | | **Lower** | | **Upper** | | ***Z*** | | ***p*** | |
| age |  | → |  | crt_no-conflict |  | a |  | 0.52358 |  | 0.13159 |  | 0.26591 |  | 0.76961 |  | 3.98 |  | < .001 |  |
| crt_no-conflict |  | → |  | fake_novel |  | b |  | -0.00110 |  | 6.40e-4 |  | -0.00232 |  | 9.24e-5 |  | -1.71 |  | 0.087 |  |
| age |  | → |  | fake_novel |  | c |  | -0.00562 |  | 0.00220 |  | -0.01001 |  | -0.00172 |  | -2.56 |  | 0.011 |  |
|  | | | | | | | | | | | | | | | | | | | |

Table S17. Results of mediation analysis with age as the independent variable, real news accuracy ratings as the dependent variable, and CRT conflict (A) and CRT no-conflict (B) as mediators, restricted to novel news.

(A) CRT conflict items as mediator

| Mediation Estimates | | | | | | | | | | | | | | | | | |
| --- | --- | --- | --- | --- | --- | --- | --- | --- | --- | --- | --- | --- | --- | --- | --- | --- | --- |
|  | | | | | | | | **95% Confidence Interval** | | | |  | | | | | |
| **Effect** | | **Label** | | **Estimate** | | **SE** | | **Lower** | | **Upper** | | **Z** | | **p** | | **% Mediation** | |
| Indirect |  | a × b |  | 0.00100 |  | 0.00130 |  | -0.00163 |  | 0.00372 |  | 0.772 |  | 0.440 |  | 7.08 |  |
| Direct |  | c |  | 0.01313 |  | 0.00276 |  | 0.00787 |  | 0.01876 |  | 4.760 |  | < .001 |  | 92.92 |  |
| Total |  | c + a × b |  | 0.01413 |  | 0.00252 |  | 0.00930 |  | 0.01914 |  | 5.603 |  | < .001 |  | 100.00 |  |
|  | | | | | | | | | | | | | | | | | |

| Path Estimates | | | | | | | | | | | | | | | | | | | |
| --- | --- | --- | --- | --- | --- | --- | --- | --- | --- | --- | --- | --- | --- | --- | --- | --- | --- | --- | --- |
|  | | | | | | | | | | | | **95% Confidence Interval** | | | |  | | | |
|  | |  | |  | | **Label** | | **Estimate** | | **SE** | | **Lower** | | **Upper** | | **Z** | | **p** | |
| age |  | → |  | crt_conflict |  | a |  | 2.5040 |  | 0.27612 |  | 1.98365 |  | 3.08006 |  | 9.069 |  | < .001 |  |
| crt_conflict |  | → |  | real_novel |  | b |  | 3.99e-4 |  | 5.08e-4 |  | -6.85e−4 |  | 0.00142 |  | 0.786 |  | 0.432 |  |
| age |  | → |  | real_novel |  | c |  | 0.0131 |  | 0.00276 |  | 0.00787 |  | 0.01876 |  | 4.760 |  | < .001 |  |
|  | | | | | | | | | | | | | | | | | | | |

(B) CRT no-conflict items as mediator

| Mediation Estimates | | | | | | | | | | | | | | | | | |
| --- | --- | --- | --- | --- | --- | --- | --- | --- | --- | --- | --- | --- | --- | --- | --- | --- | --- |
|  | | | | | | | | **95% Confidence Interval** | | | |  | | | | | |
| **Effect** | | **Label** | | **Estimate** | | **SE** | | **Lower** | | **Upper** | | **Z** | | **p** | | **% Mediation** | |
| Indirect |  | a × b |  | 1.83e-4 |  | 3.47e-4 |  | -4.57e−4 |  | 9.11e-4 |  | 0.527 |  | 0.598 |  | 1.29 |  |
| Direct |  | c |  | 0.0139 |  | 0.00267 |  | 0.00870 |  | 0.0195 |  | 5.226 |  | < .001 |  | 98.71 |  |
| Total |  | c + a × b |  | 0.0141 |  | 0.00264 |  | 0.00894 |  | 0.0196 |  | 5.361 |  | < .001 |  | 100.00 |  |
|  | | | | | | | | | | | | | | | | | |

| Path Estimates | | | | | | | | | | | | | | | | | | | |
| --- | --- | --- | --- | --- | --- | --- | --- | --- | --- | --- | --- | --- | --- | --- | --- | --- | --- | --- | --- |
|  | | | | | | | | | | | | **95% Confidence Interval** | | | |  | | | |
|  | |  | |  | | **Label** | | **Estimate** | | **SE** | | **Lower** | | **Upper** | | **Z** | | **p** | |
| age |  | → |  | crt_no-conflict |  | a |  | 0.5236 |  | 0.12972 |  | 0.26209 |  | 0.79937 |  | 4.036 |  | < .001 |  |
| crt_no-conflict |  | → |  | real_novel |  | b |  | 3.49e-4 |  | 6.26e-4 |  | -8.54e−4 |  | 0.00157 |  | 0.557 |  | 0.577 |  |
| age |  | → |  | real_novel |  | c |  | 0.0139 |  | 0.00267 |  | 0.00870 |  | 0.01946 |  | 5.226 |  | < .001 |  |
|  | | | | | | | | | | | | | | | | | | | |

Table S18. Summary statistics of media truth discernment scores per age group, restricted to repeated news items.

SD stands for standard deviations, 95% CI for 95% confidence interval, min-max for the minimum and maximum scores.

|  |  | mtd |
| --- | --- | --- |
| 11 years old | *M* (*SD*) | 0.007 (0.13) |
|  | 95% CI | [-0.01 ; 0.03] |
|  | min-max | -0.31 ; 0.30 |
|  | skewness | -0.182 |
| 12 years old | *M* (*SD*) | 0.02 (0.12) |
|  | 95% CI | [0.002 ; 0.04] |
|  | min-max | -0.30 ; 0.38 |
|  | skewness | -0.063 |
| 13 years old | *M* (*SD*) | 0.05 (0.10) |
|  | 95% CI | [0.03 ; 0.07] |
|  | min-max | -0.25 ; 0.40 |
|  | skewness | 0.218 |
| 14 years old | *M* (*SD*) | 0.10 (0.12) |
|  | 95% CI | [0.08 ; 0.13] |
|  | min-max | -0.22 ; 0.46 |
|  | skewness | 0.029 |
| Adults | *M* (*SD*) | 0.15 (0.13) |
|  | 95% CI | [0.13 ; 0.17] |
|  | min-max | -0.25 ; 0.52 |
|  | skewness | -0.052 |

Table S19. Summary statistics of mean accuracy ratings per age group, restricted to repeated news items.

SD stands for standard deviations, 95% CI for 95% confidence interval, min-max for the minimum and maximum scores.

|  |  | Real news | Fake news |
| --- | --- | --- | --- |
| 11 years old | *M* (*SD*) | 2.60 (0.456) | 2.58 (0.444) |
|  | 95% CI | [2.52 ; 2.69] | [2.50 ; 2.66] |
|  | min-max | 1.43 ; 3.50 | 1.43 ; 3.50 |
|  | skewness | -0.343 | -0.298 |
| 12 years old | *M* (*SD*) | 2.60 (0.423) | 2.52 (0.449) |
|  | 95% CI | [2.52 ; 2.68] | [2.44 ; 2.61] |
|  | min-max | 1.57 ; 3.57 | 1.50 ; 3.50 |
|  | skewness | -0.104 | 0.023 |
| 13 years old | *M* (*SD*) | 2.68 (0.399) | 2.52 (0.387) |
|  | 95% CI | [2.61 ; 2.76] | [2.44 ; 2.59] |
|  | min-max | 1.71 ; 3.71 | 1.50 ; 3.67 |
|  | skewness | - 0.108 | 0.135 |
| 14 years old | *M* (*SD*) | 2.71 (0.312) | 2.39 (0.331) |
|  | 95% CI | [2.65 ; 2.77] | [2.33 ; 2.45] |
|  | min-max | 2.00 ; 3.57 | 1.67 ; 3.36 |
|  | skewness | -0.039 | 0.394 |
| Adults | *M* (*SD*) | 2.86 (0.421) | 2.39 (0.431) |
|  | 95% CI | [2.78 ; 2.93] | [2.32 ; 2.46] |
|  | min-max | 1.42 ; 3.86 | 1.43 ; 3.57 |
|  | skewness | -0.336 | 0.372 |

Table S20. Developmental effect on the media truth discernment restricted to repeated news items.

| (A) ANOVA table | | | | | | | | | | | | | |
| --- | --- | --- | --- | --- | --- | --- | --- | --- | --- | --- | --- | --- | --- |
|  | | Sum of Squares | | *df* | | Mean Square | | *F* | | *p* | | *η²_p_* [95% CI] | |
| Age group |  | 1.75 |  | 4 |  | 0.4380 |  | 27.7 |  | < .001 |  | 0.17 [0.12; 1.00] |  |
| Residuals |  | 8.82 |  | 559 |  | 0.0158 |  |  |  |  |  |  |  |
|  | | | | | | | | | | | | | |

(B) Polynomial contrasts

|  | Estimate | se | t | p |
| --- | --- | --- | --- | --- |
| linear | 0.11869 | 0.0116 | 10.220 | < .001 |
| quadratic | 0.02237 | 0.0117 | 1.906 | 0.057 |
| cubic | -0.00404 | 0.0120 | -0.336 | 0.737 |
| quartic | -0.00428 | 0.0121 | -0.354 | 0.723 |

(C) Post-hoc analysis with holm correction

| Comparisons | Mean difference | se | df | t | p_holm_ | Cohen's d | 95% CI |
| --- | --- | --- | --- | --- | --- | --- | --- |
| 11 yo - 12 yo | -0.0183 | 0.0170 | 559 | -1.08 | 0.282 | -0.09 | [-0.26; 0.07] |
| 12 yo - 13 yo | -0.0290 | 0.0171 | 559 | -1.70 | 0.179 | -0.14 | [-0.31; 0.02] |
| 13 yo – 14 yo | -0.0512 | 0.0172 | 559 | -2.98 | < .05 | -0.25 | [-0.42; -0.09] |
| 14 yo - adults | -0.0491 | 0.0164 | 559 | -2.99 | < .05 | -0.25 | [-0.42; -0.09] |

Table S21. Developmental effect on mean accuracy ratings for real and fake news items restricted to repeated news items.

(A) ANOVA table

 Within Subjects effects

|  | | Sum of Squares | | *df* | | Mean Square | | *F* | | *p* | | *η²_p_* [95% CI] | |  |
| --- | --- | --- | --- | --- | --- | --- | --- | --- | --- | --- | --- | --- | --- | --- |
| Veracity |  | 12.43 |  | 1 |  | 12.4323 |  | 175.0 |  | < .001 |  | 0.238 [0.19; 1.00] |  | |
| Veracity ✻ Age group |  | 7.88 |  | 4 |  | 1.9709 |  | 27.7 |  | < .001 |  | 0.166 [0.12; 1.00] |  | |
| Residual |  | 39.70 |  | 559 |  | 0.0710 |  |  |  |  |  |  |  | |
|  | | | | | | | | | | | | | |  |
|  | | | | | | | | | | | | | |  |

| Between Subjects Effects | | | | | | | | | | | | | |
| --- | --- | --- | --- | --- | --- | --- | --- | --- | --- | --- | --- | --- | --- |
|  | | Sum of Squares | | *df* | | Mean Square | | *F* | | *p* | | *η²_p_* [95% CI] | |
| Age group |  | 0.802 |  | 4 |  | 0.201 |  | 0.759 |  | 0.552 |  | 0.005 [0.00; 1.00] |  |
| Residual |  | 147.683 |  | 559 |  | 0.264 |  |  |  |  |  |  |  |
| Note. Type 3 Sums of Squares | | | | | | | | | | | | | |
|  | | | | | | | | | | | | | |

(B) Post-hoc analysis with holm correction

| Age group | real news items (mean accuracy ratings) | fake news items (mean accuracy ratings) | t(df) | p _holm_ | Cohen's d | 95% CI |
| --- | --- | --- | --- | --- | --- | --- |
| 11 yo | 2.60 (0.45) | 2.58 (0.44) | 0.66 (559) | 0.511 | 0.03 | [-0.06; 0.11] |
| 12 yo | 2.60 (0.42) | 2.52 (0.44) | 2.17 (559) | 0.06 | 0.09 | [0.01; 0.17] |
| 13 yo | 2.68 (0.39) | 2.52 (0.38) | 4.59 (559) | <.001 | 0.19 | [0.11; 0.28] |
| 14 yo | 2.71 (0.31) | 2.39 (0.33) | 8.68 (559) | <.001 | 0.37 | [0.28; 0.45] |
| Adults | 2.86 (0.42) | 2.39 (0.43) | 14.22 (559) | <.001 | 0.60 | [0.51; 0.69] |

Table S22. Partial Pearson correlations controlled for age, restricted to repeated news items.

|  |  | mtd | real news | fake news | crt conflict | crt no conflict |
| --- | --- | --- | --- | --- | --- | --- |
| mtd | Pearson's *r* | — |  |  |  |  |
|  | *p*-value | — |  |  |  |  |
| real news | Pearson's *r* | 0.453*** | — |  |  |  |
|  | *p*-value | < .001 | — |  |  |  |
| fake news | Pearson's *r* | -0.489*** | 0.556*** | — |  |  |
|  | *p*-value | < .001 | < .001 | — |  |  |
| crt conflict | Pearson's *r* | 0.166*** | 0.055 | -0.101* | — |  |
|  | *p*-value | < .001 | 0.192 | 0.016 | — |  |
| crt no conflict | Pearson's *r* | 0.079* | 0.067 | -0.008 | 0.156*** | — |
|  | *p*-value | 0.061 | 0.113 | 0.843 | < .001 | — |

Note. * *p* < .05, ** *p* < .01, *** *p* < .001

Table S23. Results of mediation analysis with age as the independent variable, media truth discernment as the dependent variable, and CRT conflict (A) and CRT no-conflict (B) as mediators, restricted to repeated news items.

(A) CRT conflict items as mediator

| Mediation Estimates | | | | | | | | | | | | | | | | | |
| --- | --- | --- | --- | --- | --- | --- | --- | --- | --- | --- | --- | --- | --- | --- | --- | --- | --- |
|  | | | | | | | | **95% Confidence Interval** | | | |  | | | | | |
| **Effect** | | **Label** | | **Estimate** | | ***SE*** | | **Lower** | | **Upper** | | ***Z*** | | ***p*** | | **% Mediation** | |
| Indirect |  | a × b |  | 0.00171 |  | 4.78e-4 |  | 8.19e-4 |  | 0.00267 |  | 3.58 |  | < .001 |  | 25.0 |  |
| Direct |  | c |  | 0.00515 |  | 9.87e-4 |  | 0.00328 |  | 0.00704 |  | 5.22 |  | < .001 |  | 75.0 |  |
| Total |  | c + a × b |  | 0.00686 |  | 9.12e-4 |  | 0.00510 |  | 0.00870 |  | 7.53 |  | < .001 |  | 100.0 |  |
|  | | | | | | | | | | | | | | | | | |

| Path Estimates | | | | | | | | | | | | | | | | | | | |
| --- | --- | --- | --- | --- | --- | --- | --- | --- | --- | --- | --- | --- | --- | --- | --- | --- | --- | --- | --- |
|  | | | | | | | | | | | | **95% Confidence Interval** | | | |  | | | |
|  | |  | |  | | **Label** | | **Estimate** | | ***SE*** | | **Lower** | | **Upper** | | ***Z*** | | ***p*** | |
| age |  | → |  | crt_conflict |  | a |  | 2.50402 |  | 0.275 |  | 1.98446 |  | 3.07738 |  | 9.10 |  | < .001 |  |
| crt_conflict |  | → |  | mdt_repeated1 |  | b |  | 6.84e-4 |  | 1.81e-4 |  | 3.20e-4 |  | 0.00106 |  | 3.77 |  | < .001 |  |
| age |  | → |  | mdt_repeated1 |  | c |  | 0.00515 |  | 9.87e-4 |  | 0.00328 |  | 0.00704 |  | 5.22 |  | < .001 |  |
|  | | | | | | | | | | | | | | | | | | | |

(B) CRT no-conflict items as mediator

| Mediation Estimates | | | | | | | | | | | | | | | | | |
| --- | --- | --- | --- | --- | --- | --- | --- | --- | --- | --- | --- | --- | --- | --- | --- | --- | --- |
|  | | | | | | | | **95% Confidence Interval** | | | |  | | | | | |
| **Effect** | | **Label** | | **Estimate** | | ***SE*** | | **Lower** | | **Upper** | | ***Z*** | | ***p*** | | **% Mediation** | |
| Indirect |  | a × b |  | 2.11e-4 |  | 1.18e-4 |  | -8.23e−6 |  | 4.46e-4 |  | 1.78 |  | 0.075 |  | 3.07 |  |
| Direct |  | c |  | 0.00665 |  | 8.92e-4 |  | 0.00505 |  | 0.00853 |  | 7.46 |  | < .001 |  | 96.93 |  |
| Total |  | c + a × b |  | 0.00686 |  | 8.99e-4 |  | 0.00529 |  | 0.00876 |  | 7.64 |  | < .001 |  | 100.00 |  |
|  | | | | | | | | | | | | | | | | | |

| Path Estimates | | | | | | | | | | | | | | | | | | | |
| --- | --- | --- | --- | --- | --- | --- | --- | --- | --- | --- | --- | --- | --- | --- | --- | --- | --- | --- | --- |
|  | | | | | | | | | | | | **95% Confidence Interval** | | | |  | | | |
|  | |  | |  | | **Label** | | **Estimate** | | ***SE*** | | **Lower** | | **Upper** | | ***Z*** | | ***p*** | |
| age |  | → |  | crt_no-conflict |  | a |  | 0.52358 |  | 0.129 |  | 0.27191 |  | 0.77858 |  | 4.06 |  | < .001 |  |
| crt_no-conflict |  | → |  | mdt_repeated1 |  | b |  | 4.02e-4 |  | 2.10e-4 |  | -1.38e−5 |  | 7.94e-4 |  | 1.92 |  | 0.055 |  |
| age |  | → |  | mdt_repeated1 |  | c |  | 0.00665 |  | 8.92e-4 |  | 0.00505 |  | 0.00853 |  | 7.46 |  | < .001 |  |
|  | | | | | | | | | | | | | | | | | | | |

Table S24. Results of mediation analysis with age as the independent variable, fake news accuracy ratings as the dependent variable, and CRT conflict (A) and CRT no-conflict (B) as mediators, restricted to repeated news items.

(A) CRT conflict items as mediator

| Mediation Estimates | | | | | | | | | | | | | | | | | |
| --- | --- | --- | --- | --- | --- | --- | --- | --- | --- | --- | --- | --- | --- | --- | --- | --- | --- |
|  | | | | | | | | **95% Confidence Interval** | | | |  | | | | | |
| **Effect** | | **Label** | | **Estimate** | | ***SE*** | | **Lower** | | **Upper** | | ***Z*** | | ***p*** | | **% Mediation** | |
| Indirect |  | a × b |  | -0.00335 |  | 0.00147 |  | -0.00640 |  | -4.82e−4 |  | -2.28 |  | 0.023 |  | 48.8 |  |
| Direct |  | c |  | -0.00351 |  | 0.00310 |  | -0.00969 |  | 0.00231 |  | -1.13 |  | 0.257 |  | 51.2 |  |
| Total |  | c + a × b |  | -0.00686 |  | 0.00276 |  | -0.01195 |  | -0.00136 |  | -2.49 |  | 0.013 |  | 100.0 |  |
|  | | | | | | | | | | | | | | | | | |

| Path Estimates | | | | | | | | | | | | | | | | | | | |
| --- | --- | --- | --- | --- | --- | --- | --- | --- | --- | --- | --- | --- | --- | --- | --- | --- | --- | --- | --- |
|  | | | | | | | | | | | | **95% Confidence Interval** | | | |  | | | |
|  | |  | |  | | **Label** | | **Estimate** | | ***SE*** | | **Lower** | | **Upper** | | ***Z*** | | ***p*** | |
| age |  | → |  | crt_conflict |  | a |  | 2.50402 |  | 0.26914 |  | 2.00654 |  | 3.05104 |  | 9.30 |  | < .001 |  |
| crt_conflict |  | → |  | fake_repeated |  | b |  | -0.00134 |  | 5.74e-4 |  | -0.00252 |  | -2.04e−4 |  | -2.33 |  | 0.020 |  |
| age |  | → |  | fake_repeated |  | c |  | -0.00351 |  | 0.00310 |  | -0.00969 |  | 0.00231 |  | -1.13 |  | 0.257 |  |
|  | | | | | | | | | | | | | | | | | | | |

(B) CRT no-conflict items as mediator

| Mediation Estimates | | | | | | | | | | | | | | | | | |
| --- | --- | --- | --- | --- | --- | --- | --- | --- | --- | --- | --- | --- | --- | --- | --- | --- | --- |
|  | | | | | | | | **95% Confidence Interval** | | | |  | | | | | |
| **Effect** | | **Label** | | **Estimate** | | ***SE*** | | **Lower** | | **Upper** | | ***Z*** | | ***p*** | | **% Mediation** | |
| Indirect |  | a × b |  | -7.18e−5 |  | 3.74e-4 |  | -7.63e−4 |  | 7.52e-4 |  | -0.192 |  | 0.848 |  | 1.05 |  |
| Direct |  | c |  | -0.00679 |  | 0.00286 |  | -0.0126 |  | -0.00159 |  | -2.371 |  | 0.018 |  | 98.95 |  |
| Total |  | c + a × b |  | -0.00686 |  | 0.00281 |  | -0.0125 |  | -0.00161 |  | -2.440 |  | 0.015 |  | 100.00 |  |
|  | | | | | | | | | | | | | | | | | |

| Path Estimates | | | | | | | | | | | | | | | | | | | |
| --- | --- | --- | --- | --- | --- | --- | --- | --- | --- | --- | --- | --- | --- | --- | --- | --- | --- | --- | --- |
|  | | | | | | | | | | | | **95% Confidence Interval** | | | |  | | | |
|  | |  | |  | | **Label** | | **Estimate** | | ***SE*** | | **Lower** | | **Upper** | | ***Z*** | | ***p*** | |
| age |  | → |  | crt_no-conflict |  | a |  | 0.52358 |  | 0.13656 |  | 0.23874 |  | 0.77641 |  | 3.834 |  | < .001 |  |
| crt_no-conflict |  | → |  | fake_repeated |  | b |  | -1.37e−4 |  | 6.97e-4 |  | -0.00147 |  | 0.00126 |  | -0.197 |  | 0.844 |  |
| age |  | → |  | fake_repeated |  | c |  | -0.00679 |  | 0.00286 |  | -0.01263 |  | -0.00159 |  | -2.371 |  | 0.018 |  |
|  | | | | | | | | | | | | | | | | | | | |

Table S25. Results of mediation analysis with age as the independent variable, real news accuracy ratings as the dependent variable, and CRT conflict (A) and CRT no-conflict (B) as mediators, restricted to repeated news.

(A) CRT conflict items as mediator

| Mediation Estimates | | | | | | | | | | | | | | | | | |
| --- | --- | --- | --- | --- | --- | --- | --- | --- | --- | --- | --- | --- | --- | --- | --- | --- | --- |
|  | | | | | | | | **95% Confidence Interval** | | | |  | | | | | |
| **Effect** | | **Label** | | **Estimate** | | ***SE*** | | **Lower** | | **Upper** | | ***Z*** | | ***p*** | | **% Mediation** | |
| Indirect |  | a × b |  | 0.00179 |  | 0.00142 |  | -0.00102 |  | 0.00453 |  | 1.26 |  | 0.209 |  | 13.0 |  |
| Direct |  | c |  | 0.01194 |  | 0.00340 |  | 0.00521 |  | 0.01847 |  | 3.51 |  | < .001 |  | 87.0 |  |
| Total |  | c + a × b |  | 0.01372 |  | 0.00298 |  | 0.00817 |  | 0.01955 |  | 4.60 |  | < .001 |  | 100.0 |  |
|  | | | | | | | | | | | | | | | | | |

| Path Estimates | | | | | | | | | | | | | | | | | | | |
| --- | --- | --- | --- | --- | --- | --- | --- | --- | --- | --- | --- | --- | --- | --- | --- | --- | --- | --- | --- |
|  | | | | | | | | | | | | **95% Confidence Interval** | | | |  | | | |
|  | |  | |  | | **Label** | | **Estimate** | | ***SE*** | | **Lower** | | **Upper** | | ***Z*** | | ***p*** | |
| age |  | → |  | crt_conflict |  | a |  | 2.5040 |  | 0.26945 |  | 2.01700 |  | 3.03287 |  | 9.29 |  | < .001 |  |
| crt_conflict |  | → |  | real_repeated |  | b |  | 7.13e-4 |  | 5.62e-4 |  | -4.13e−4 |  | 0.00178 |  | 1.27 |  | 0.204 |  |
| age |  | → |  | real_repeated |  | c |  | 0.0119 |  | 0.00340 |  | 0.00521 |  | 0.01847 |  | 3.51 |  | < .001 |  |
|  | | | | | | | | | | | | | | | | | | | |

(B) CRT no-conflict items as mediator

| Mediation Estimates | | | | | | | | | | | | | | | | | |
| --- | --- | --- | --- | --- | --- | --- | --- | --- | --- | --- | --- | --- | --- | --- | --- | --- | --- |
|  | | | | | | | | **95% Confidence Interval** | | | |  | | | | | |
| **Effect** | | **Label** | | **Estimate** | | ***SE*** | | **Lower** | | **Upper** | | ***Z*** | | ***p*** | | **% Mediation** | |
| Indirect |  | a × b |  | 5.60e-4 |  | 3.95e-4 |  | -1.52e−4 |  | 0.00142 |  | 1.42 |  | 0.157 |  | 4.08 |  |
| Direct |  | c |  | 0.0132 |  | 0.00290 |  | 0.00756 |  | 0.01907 |  | 4.53 |  | < .001 |  | 95.92 |  |
| Total |  | c + a × b |  | 0.0137 |  | 0.00288 |  | 0.00821 |  | 0.01958 |  | 4.77 |  | < .001 |  | 100.00 |  |
|  | | | | | | | | | | | | | | | | | |

| Path Estimates | | | | | | | | | | | | | | | | | | | |
| --- | --- | --- | --- | --- | --- | --- | --- | --- | --- | --- | --- | --- | --- | --- | --- | --- | --- | --- | --- |
|  | | | | | | | | | | | | **95% Confidence Interval** | | | |  | | | |
|  | |  | |  | | **Label** | | **Estimate** | | ***SE*** | | **Lower** | | **Upper** | | ***Z*** | | ***p*** | |
| age |  | → |  | crt_no-conflict |  | a |  | 0.52358 |  | 0.13866 |  | 0.24379 |  | 0.77864 |  | 3.78 |  | < .001 |  |
| crt_no-conflict |  | → |  | real_repeated |  | b |  | 0.00107 |  | 6.81e-4 |  | -2.76e−4 |  | 0.00234 |  | 1.57 |  | 0.116 |  |
| age |  | → |  | real_repeated |  | c |  | 0.01316 |  | 0.00290 |  | 0.00756 |  | 0.01907 |  | 4.53 |  | < .001 |  |
|  | | | | | | | | | | | | | | | | | | | |

Table S26. Summary statistics of illusory truth effect (ite) scores per age group.

SD stands for standard deviations, 95% CI for 95% confidence interval, min-max for the minimum and maximum scores.

|  |  | ite |
| --- | --- | --- |
| 11 years old | *M* (*SD*) | 0.045 (0.09) |
|  | 95% CI | [0.027 ; 0.064] |
|  | min-max | -0.190 ; 0.270 |
|  | skewness | -0.01 |
| 12 years old | *M* (*SD*) | 0.042 (0.09) |
|  | 95% CI | [0.023 ; 0.060] |
|  | min-max | -0.147 ; 0.337 |
|  | skewness | 0.383 |
| 13 years old | *M* (*SD*) | 0.065 (0.109) |
|  | 95% CI | [0.044 ; 0.086] |
|  | min-max | -0.193 ; 0.570 |
|  | skewness | 1.56 |
| 14 years old | *M* (*SD*) | 0.069 (0.09) |
|  | 95% CI | [0.051 ; 0.087] |
|  | min-max | -0.133 ; 0.440 |
|  | skewness | 0.677 |
| Adults | *M* (*SD*) | 0.046 (0.09) |
|  | 95% CI | [0.029 ; 0.062] |
|  | min-max | -0.147 ; 0.300 |
|  | skewness | 0.320 |

Table S27. Summary statistics of accuracy ratings per age group in the illusory truth effect paradigm.

SD stands for standard deviations, 95% CI for 95% confidence interval, min-max for the minimum and maximum scores.

|  |  | Novel | Familiarised |
| --- | --- | --- | --- |
| 11 years old | *M* (*SD*) | 2.46 (0.34) | 2.59 (0.40) |
|  | 95% CI | [2.39 ; 2.52] | [2.52 ; 2.67] |
|  | min-max | 1.50 - 3.18 | 1.61 - 3.38 |
|  | skewness | -0.15 | -0.32 |
| 12 years old | *M* (*SD*) | 2.44 (0.36) | 2.57 (0.39) |
|  | 95% CI | [2.37 ; 2.51] | [2.49 ; 2.64] |
|  | min-max | 1.57 - 3.25 | 1.57 - 3.50 |
|  | skewness | -0.02 | -0.14 |
| 13 years old | *M* (*SD*) | 2.41 (0.33) | 2.60 (0.36) |
|  | 95% CI | [2.34 ; 2.47] | [2.53 ; 2.67] |
|  | min-max | 1.67 - 3.13 | 1.79 - 3.61 |
|  | skewness | 0.10 | -0.01 |
| 14 years old | *M* (*SD*) | 2.34 (0.30) | 2.55 (0.26) |
|  | 95% CI | [2.29 ; 2.40] | [2.50 ; 2.60] |
|  | min-max | 1.50 - 3.11 | 1.93 - 3.43 |
|  | skewness | -0.10 | 0.16 |
| Adults | *M* (*SD*) | 2.49 (0.30) | 2.62 (0.37) |
|  | 95% CI | [2.43 ; 2.54] | [2.56 ; 2.69] |
|  | min-max | 1.79 - 3.35 | 1.50 - 3.71 |
|  | skewness | 0.37 | 0.08 |

Table S28. Developmental effect on the illusory truth effect scores.

ANOVA table

|  | | Sum of Squares | | df | | Mean Square | | F | | p | | η²_p_ [95% CI] | |
| --- | --- | --- | --- | --- | --- | --- | --- | --- | --- | --- | --- | --- | --- |
| Age group |  | 0.0716 |  | 4 |  | 0.01790 |  | 1.82 |  | 0.123 |  | 0.22 [0.17, 1.00] |  |
| Residuals |  | 5.4931 |  | 559 |  | 0.00983 |  |  |  |  |  |  |  |
|  | | | | | | | | | | | | | |

Table S29. Demographic table

Number of participants, mean age in years, and gender in each age group. SD stands for standard deviation. The column “gender” represents the number of female (f), male (m) and other (o) in each group.

|  | N | M (SD) | Gender (f,m,o) |
| --- | --- | --- | --- |
| 11 years old | 110 | 11.25 (0.48) | 56, 49, 5 |
| 12 years old | 108 | 12.3 (0.45) | 62, 46, 0 |
| 13 years old | 109 | 13.1 (0.54) | 52, 57, 0 |
| 14 years old | 105 | 14.3 (0.46) | 55, 50, 0 |
| Adults | 132 | 27 (5.16) | 62, 70, 0 |

Table S30. Word count analysis in headlines titles for fake and real news items

| ANOVA | | | | | | | | | | | |
| --- | --- | --- | --- | --- | --- | --- | --- | --- | --- | --- | --- |
|  | | Sum of Squares | | *df* | | Mean Square | | *F* | | *p* | |
| Veracity |  | 3.50 |  | 1 |  | 3.50 |  | 0.311 |  | 0.579 |  |
| Residuals |  | 607.86 |  | 54 |  | 11.26 |  |  |  |  |  |
|  | | | | | | | | | | | |

Table S31. Word count analysis in headlines catchphrases for fake and real news items

| ANOVA | | | | | | | | | | | |
| --- | --- | --- | --- | --- | --- | --- | --- | --- | --- | --- | --- |
|  | | Sum of Squares | | *df* | | Mean Square | | *F* | | *p* | |
| Veracity |  | 12.1 |  | 1 |  | 12.1 |  | 0.310 |  | 0.580 |  |
| Residuals |  | 2102.8 |  | 54 |  | 38.9 |  |  |  |  |  |
|  | | | | | | | | | | | |
